# Three-Dimensional Model of Sub-Plasmalemmal Ca^2+^ Microdomains Evoked by T Cell Receptor/CD3 Complex Stimulation

**DOI:** 10.3389/fmolb.2022.811145

**Published:** 2022-02-23

**Authors:** Diana Gil, Björn-Philipp Diercks, Andreas H. Guse, Geneviève Dupont

**Affiliations:** ^1^ The Calcium Signalling Group, Department of Biochemistry and Molecular Cell Biology, University Medical Center Hamburg-Eppendorf, Hamburg, Germany; ^2^ Unit of Theoretical Chronobiology, Faculté des Sciences CP231, Université Libre de Bruxelles (ULB), Brussels, Belgium

**Keywords:** T cells, ER-PM junctions, ryanodine receptors, NAADP, COMSOL, computational model, store operated calcium entry, ca^2+^ signalling

## Abstract

Ca^2+^ signalling plays an essential role in T cell activation, which is a key step to start an adaptive immune response. During the transition from a quiescent to a fully activated state, Ca^2+^ microdomains of reduced spatial and temporal extents develop in the junctions between the plasma membrane and the endoplasmic reticulum (ER). These microdomains rely on Ca^2+^ entry from the extracellular medium, via the ORAI1/STIM1/STIM2 system that mediates store operated Ca^2+^ entry Store operated calcium entry. The mechanism leading to local store depletion and subsequent Ca^2+^ entry depends on the activation state of the cells. The initial, smaller microdomains are triggered by D-*myo*-inositol 1,4,5-trisphosphate (IP_3_) signalling in response to T cell adhesion. T cell receptor (TCR)/CD3 stimulation then initiates nicotinic acid adenine dinucleotide phosphate signalling, which activates ryanodine receptors (RYR). We have recently developed a mathematical model to elucidate the spatiotemporal Ca^2+^ dynamics of the microdomains triggered by IP_3_ signalling in response to T cell adhesion (Gil et al., 2021). This reaction-diffusion model describes the evolution of the cytosolic and endoplasmic reticulum Ca^2+^ concentrations in a three-dimensional ER-PM junction and was solved using COMSOL Multiphysics. Modelling predicted that adhesion-dependent microdomains result from the concerted activity of IP_3_ receptors and pre-formed ORAI1-STIM2 complexes. In the present study, we extend this model to include the role of RYRs rapidly after TCR/CD3 stimulation. The involvement of STIM1, which has a lower K_D_ for Ca^2+^ than STIM2, is also considered. Detailed 3D spatio-temporal simulations show that these Ca^2+^ microdomains rely on the concerted opening of ∼7 RYRs that are simultaneously active in response to the increase in NAADP induced by T cell stimulation. Opening of these RYRs provoke a local depletion of ER Ca^2+^ that triggers Ca^2+^ flux through the ORAI1 channels. Simulations predict that RYRs are most probably located around the junction and that the increase in junctional Ca^2+^ concentration results from the combination between diffusion of Ca^2+^ released through the RYRs and Ca^2+^ entry through ORAI1 in the junction. The computational model moreover provides a tool allowing to investigate how Ca^2+^ microdomains occur, extend and interact in various states of T cell activation.

## Introduction

Calcium signaling plays a crucial role in the activation of T cells and the adaptative immune response. In particular, it controls transcriptional activation, proliferation, differentiation or secretion of cytokines (Feske, 2007; Trebak & Kinet, 2019). Increases of the free cytosolic Ca^2+^ concentration rely on Ca^2+^ release from the endoplasmic reticulum (ER) and on Ca^2+^ entry from the extracellular medium. Mobilization of internal Ca^2+^ follows the increase in d-*myo*-inositol 1,4,5-trisphosphate (IP_3_) and in nicotinic acid adenine dinucleotide phosphate (NAADP), via IP_3_ receptors (IP_3_R) and type 1 ryanodine receptor (RYR1), respectively (Streb et al., 1983; Wolf et al., 2015). Ca^2+^ entry relies on the ORAI/STIM system that allows Ca^2+^ entry in the cytosol, at a rate that is regulated by the concentration of Ca^2+^ in lumen of the ER (Putney, 2009). When Ca^2+^ dissociates from the Ca^2+^ sensors stromal interaction molecules 1 (STIM1) and 2 (STIM2) located in the ER membrane, STIM molecules aggregate and move to so-called “junctional spaces”. These regions correspond to the narrow cytosolic spaces between the ER and PM membranes, at locations where these membranes are separated by distances smaller than 20 nm. There, STIM molecules can recruit ORAI1 to form Ca^2+^ channels allowing Ca^2+^ to enter into the cytoplasm. This process is known as capacitative or store operated Ca^2+^ entry (SOCE). The relation between SOCE and ER Ca^2+^ concentration is nonlinear, with a K_D_ for half activation of the order of 200 μM when it depends on the dissociation of Ca^2+^ from STIM1 and of 400 μM when it depends on the dissociation of Ca^2+^ from STIM2 (Stathopulos et al., 2006; Brandman et al., 2007; Luik et al., 2008).

Upon TCR/CD3 stimulation, second messengers create a substantial release of Ca^2+^ from the ER which, together with the resulting activation of SOCE, leads to a rise in the free cytosolic Ca^2+^ concentration in the whole T cell. This global Ca^2+^ increase contrasts with the locally restricted, sub-plasmalemmal Ca^2+^ increases of short duration (∼50 ms) that can be observed as a consequence of adhesive interactions (Weiss and Diercks, unpublished results), or in the first seconds following TCR/CD3 stimulation (Wolf et al., 2015; Diercks et al., 2018). The two types of events are known as Ca^2+^ microdomains and have similar spatio-temporal characteristics. Yet, they have different molecular origins. The adhesion dependent Ca^2+^ microdomains rely on a pathway involving focal adhesion kinase (FAK), phospholipase C (PLC) and IP_3_Rs (Weiss and Diercks, unpublished results). Due to IP_3_R-mediated Ca^2+^ release and subsequent SOCE, they also rely on ORAI1-mediated Ca^2+^ entry. Computational simulations of the interplay between ORAI1 and IP_3_R in a 3D configuration simulating an ER-PM junction have confirmed that the local depletion of ER Ca^2+^ created by the opening of a few IP_3_R can trigger the opening of ORAI1 in the junction, even in conditions of a full ER (Gil et al., 2021). Interestingly, these non-TCR/CD3 dependent Ca^2+^ microdomains require the existence of pre-formed complexes of ORAI1 and STIM2 that were demonstrated experimentally (Diercks et al., 2018). Ca^2+^ microdomains characterized by somewhat larger amplitude (340 ± 11 nM *vs* 290 ± 12 nM) are observed during the first ∼15 s following TCR/CD3 stimulation (Wolf et al., 2015; Diercks et al., 2018; reviewed in Guse et al., 2021). As the non-TCR/CD3 dependent microdomains, these signals also involve ORAI1, but in addition they require NAADP signaling and RYR1 dependent Ca^2+^ release from ER (Wolf et al., 2015; Diercks et al., 2018). As another difference, at this stage, pre-formed ORAI1/STIM complexes involve both STIM1 and STIM2 isoforms (Ahmad et al., 2021).

Ca^2+^ microdomains represent a crucial step for the successful activation of T cells. Reported durations of the signals triggered by cell adhesion and by formation of NAADP in the first seconds upon TCR/CD3 stimulation are 44 ± 4 ms and 64 ± 3 ms, respectively (Diercks et al., 2018). They extend on 0.216 ± 0.004 μm^2^. Because of these limited temporal and spatial extents, the investigation of Ca^2+^ microdomains is technically limited by the resolution of the microscopic imaging system used. The spatial and temporal resolution of the imaging system used to characterize T cell Ca^2+^ microdomains is approx. 368 nm and 20–25 ms, respectively (Wolf et al., 2015). Mathematical modelling thus represents a useful complementary tool to investigate their molecular origin, in the line of the numerous studies devoted to small scale Ca^2+^ events (Solovey et al., 2008; Swaminathan et al., 2009; Thul et al., 2009; Rückl & Rüdiger, 2016; Walker et al., 2017).

In a previous study (Gil et al., 2021), we adapted the realistic three-dimensional mathematical description of the ER-PM junction proposed by McIvor et al. (2018) to simulate adhesion-dependent Ca^2+^ microdomains arising in T cells. This model describes Ca^2+^ dynamics in a confined 3D configuration corresponding to a junctional cytosolic space and the adjacent sub-PM ER, taking into account Ca^2+^ influx through ORAI1 and IP_3_R, Ca^2+^ pumping into the ER through SERCA, and diffusion within the cytosolic and ER compartment. The IP_3_Rs are supposed to be located close to the junctional space (Thillaiappan et al., 2017). Simulations using COMSOL Multiphysics showed that the spontaneous activity of ∼3 IP_3_Rs create a local depletion of ER Ca^2+^ that suffices to trigger the opening of ORAI1 channels located in the junction and thus, the onset of a microdomain. Because of the presence of pre-formed complexes of ORAI1 and STIM2 in unstimulated cells, opening of ORAI1 indeed rapidly follows the dissociation of Ca^2+^ from STIM2. Predictions of this model are in agreement with recent observations in HEK293 cells reporting that constitutive STIM2 clusters in ER-PM junctions sense decreases in local ER Ca^2+^ mediated by IP_3_Rs (Ahmad et al., 2021). Moreover, IP_3_R channel activity near the junctions was shown to favour STIM2 clustering in the junction.

In this study, we modified our previous model of the T cell junctions to address the molecular mechanism that underlies the TCR/CD3-evoked and NAADP and RYR-dependent Ca^2+^ microdomains occurring in the first ∼15 s that follow TCR/CD3 stimulation. We first used modeling to find out whether RYR1 are located inside or around the ER-PM junctions. The analysis was based on comparisons between simulated and experimental results both in WT and ORAI1^−/−^ T cells. The next issue related to the number of RYR1 involved in the formation of the junctional Ca^2+^ microdomains, which cannot be directly inferred from experimental observations. By contrast, modeling can determine the number of RYR1 that must open simultaneously to create the local depletion of ER Ca^2+^ triggering the appropriate level of SOCE activation. Conclusions about this number were next validated by an independent estimation of the increase in the RYR1 open probability triggered by the NAADP formation in TCR/CD3 stimulated T cells. Finally, we took advantage of the great flexibility provided by computational modeling to investigate the respective roles played by the ER Ca^2+^ channels (IP_3_R or RYR) and the Ca^2+^ sensors (STIM1 and STIM2) in shaping the characteristics of the Ca^2+^ microdomains created by the openings of the related ORAI1 channels. This analysis allowed us to propose a unifying description of the molecular mechanism underlying T cells Ca^2+^ microdomains from adhesion to early TCR/CD3 stimulation.

### Description of the Mathematical Model

**FIGURE 1:**
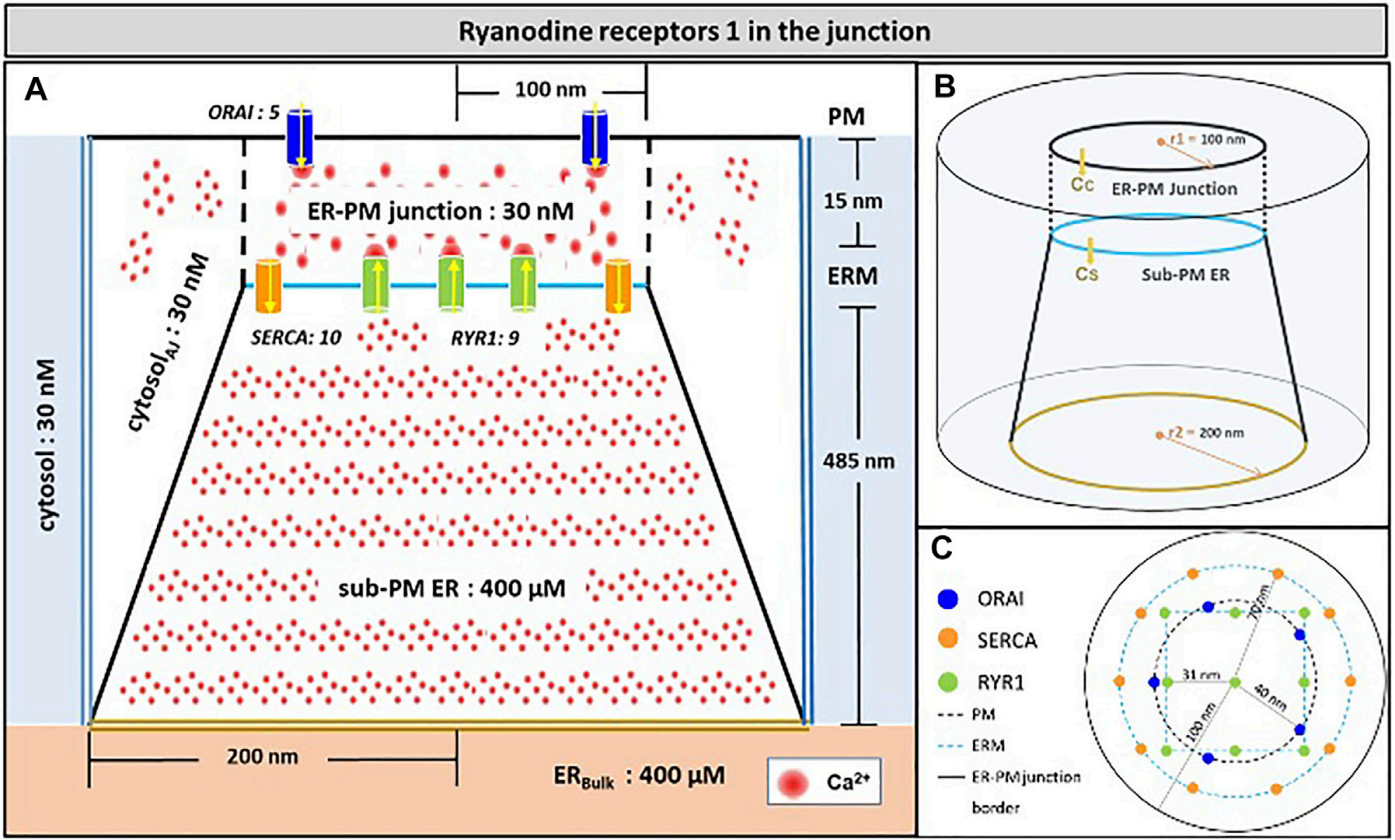
Schematic representation of the model geometry of the ER-PM junction and sub-PM ER used to investigate the origin of the Ca^2+^ microdomains in T cells with nine RYR1 inside the junction **(A)** Frontal diagram showing the dimensions of the cone that represents the sub-PM ER, of the junction and of the portion of the cytosol considered in the simulations. ORAI1 channels are in blue, SERCA pumps in orange and RYR1 in green. Plain lines represent membrane boundaries; dashed lines, fictitious limits between the junction and the cytosol and double lines indicate the limits of the simulated system. The resting Ca^2+^ concentrations considered as initial conditions and boundary conditions in the two compartments are indicated. **(B)** 3D view of the model geometry **(C)** Upper view of the positions of the ORAI1 channels on the PM, in blue, and of the SERCA pumps and RYR1 (in a chessboard manner) on the ERM, in orange and green respectively. Not to scale. This geometry is based on McIvor et al. (2018). See text for details.

Because the model was fully described in Gil et al. (2020), we provide a concise description of its main features in this section. The spatial geometry is shown in Figure 1. The junction is a 15 nm-wide (Wu et al., 2006; Hogan, 2015) three-dimensional space between the PM and an ER portion located close to it. The junction communicates with the adjacent cytoplasm, a portion of which is modelled explicitly. The free cytosolic Ca^2+^ concentration, defined by *C*_*C*_, is initially set at 30 nM (Diercks et al., 2018). In the rest of the cytoplasm, which is not modelled explicitly, *C*_*C*_ is fixed at this same value. Similarly, the evolution of ER Ca^2+^ concentration (*C*_*S*_) is simulated in the sub-PM ER, which is in contact with the bulk of the ER where Ca^2+^ concentration is fixed at 400 μM (Lewis, 2011). The PM portion located in the junction contains five ORAI channels and the ER membrane, 10 SERCA and nine RYR1s as reported (Hogan, 2015; McIvor et al., 2018; Jayasinghe et al., 2018; Yin et al., 2008). Seen from above (Figure 1C), SERCA pumps form a ring surrounding ORAI1 channels and RYR1s are arranged on a square lattice. Given the large size of these channels (Lanner et al., 2010), the 31 nm distance between the pores of the channels considered in Figure 1 corresponds to a close packing of RYR1.

Membranes, schematized as simple full lines in Figure 1A, correspond to no flux boundary conditions, except across channels and pumps where corresponding fluxes are simulated. The flux through ORAI channels is given by

$$J_{ORAI}=f\left(C_{S}^{loc}\right)\frac{I_{ORAI}}{F\cdot z\cdot A_{o}}$$ (1)

with *I*_*ORAI*_ the maximal single channel current, *F* the Faraday constant, *z* the charge of a Ca^2+^ ion and *A*_*O*_ the surface of the channel pore. $f\left(C_{S}^{loc}\right)$ is a function of the average local concentration of ER Ca^2+^ around the pore of the closest RYR1s. This step-wise function determines the level of ORAI1 activation that can take four values depending on the amount of bound Ca^2+^-free STIM. In the first phase after TCR/CD3 stimulation of T lymphocytes, preformed complexes of ORAI1, STIM1 and STIM2 have been detected by FRET experiments and super-resolution microscopy (Weiss and Diercks, unpublished results). We thus consider the activation of ORAI1 by heterotetramers of STIM1 and STIM2 (STIM1/2) and modified $f\left(C_{S}^{loc}\right)$ accordingly. See Supplementary Information for a detailed explanation.

In the ER membrane, Ca^2+^ flux from the ER to the cytosol through the RYR is given by

$$J_{RyR}=\frac{I_{RyR}}{F\cdot z\cdot A_{RyR}}\cdot \frac{\left(C_{s}-C_{C}\right)}{\left(C_{s,0}-C_{C,0}\right)}$$ (2)

with *I*_*RYR*_ the current through the RYR, which takes the value of 0.35 pA (Guo et al., 2012). *A*_*RYR*_ is the surface of the channel pore. The second factor in Eq. (2) allows to scale the current to take the actual gradient across the channel pore into account, where *C*_*S,0*_ and *C*_*C,0*_ represent resting concentrations of Ca^2+^ in the ER and in the cytosol (Mazel et al., 2009).

Finally, SERCA pumps are considered as bidirectional as in McIvor et al. (2018) and described by

$$J_{SERCA}=\frac{Q}{A_{S}}*V_{max}*\left[\frac{{\left(\frac{C_{C}}{K_{F}}\right)}^{n2}-{\left(\frac{C_{S}}{K_{R}}\right)}^{n2}}{1+{\left(\frac{C_{C}}{K_{F}}\right)}^{n2}+{\left(\frac{C_{S}}{K_{R}}\right)}^{n2}}\right]$$ (3)

with *V*_*max*_ its maximal velocity, *n2* the Hill coefficient and *K*_*F*_ and *K*_*R*_ the pump affinity for cytosolic (*C*_*C*_) and ER (*C*_*S*_) calcium, respectively. *A*_*s*_ is the surface of the pore and *Q* is a temperature coefficient initially introduced by McIvor et al. (2018). To approximate the partial differential equations (PDE), we used the finite element method (FEM) and simulation software COMSOL Multiphysics 5.5 (http://www.comsol.com), more specifically the Transport of Diluted Species interface that is used to compute the concentration field of a dilute solute in a solvent. We chose a backward differentiation formula (BDF) to compute the time steps with a relative tolerance of 0.005 that controls the relative error in each step. The system is solved using the iterative linear solver GMRES (Generalized Minimum Residual). For further details regarding the system discretization and the use of COMSOL Multiphysics, please refer to the authors.

## Results

### Ca^2+^ Microdomains Simulated by the Opening of Type 1 Ryanodine Receptors Localized in the ER-PM Junction do Not Rely on ORAI1 Opening

Upon TCR/CD3 stimulation, NAADP-evoked Ca^2+^ release through RYR1 acts in concert with Ca^2+^ entry through ORAI1/STIM complexes to create Ca^2+^ microdomains. These microdomains last for 64 ± 3 ms and reach amplitudes of 340 ± 11 nM (Diercks et al., 2018). Although it is known that ORAI1 and STIM are arranged in pre-formed complexes in the ER-PM junctions of T cells, the exact location of the RYR1s responsible for the decrease in [Ca^2+^]_ER_ in the sub-PM ER remains to be determined. As described in the presentation of the model and schematized in Figure 1, in the model we first considered that RYR1s are located in the junction, facing the PM as in cardiac dyadic clefts (Jones et al., 2018). In this section, we evaluated if this arrangement allows to reproduce experimental observations.

**FIGURE 2:**
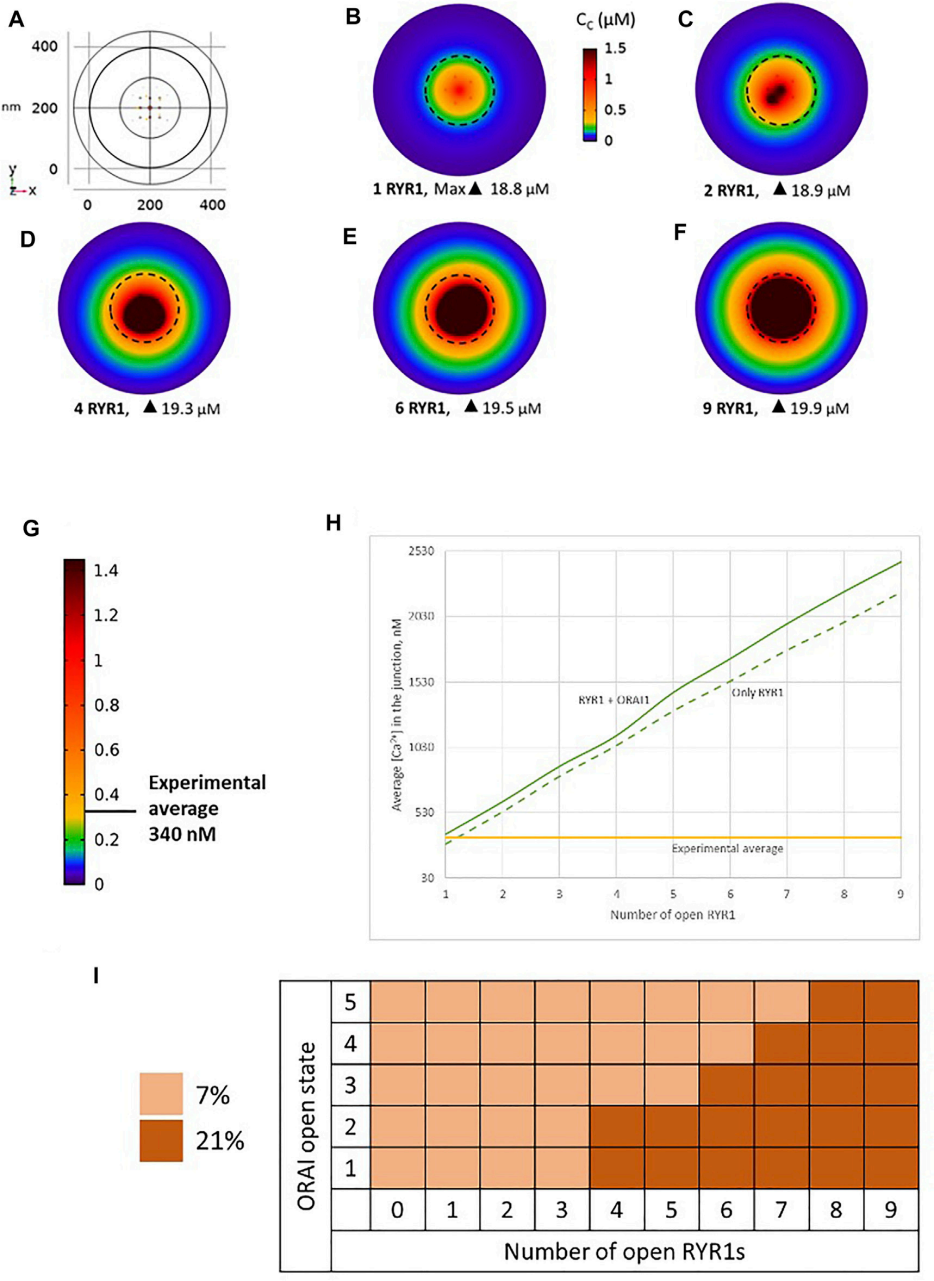
Simulated Ca^2+^ microdomains resulting from the opening of RYR1 inside the junctions, which in turn induces the opening of ORAI1 channels in the junctions as a result of local depletion of ER Ca^2+^ **(A)** Upper view of the arrangement of the ORAI1 channels on the PM of the junction (yellow dots) and of the RYR1 on the ERM (red dots) using COMSOL **(B–F)** Steady-state Ca^2+^ profiles in the junction when opening 1, 2, 4, 6 and 9 RYR1 simultaneously **(B)** to **(F)** respectively. Shown are the profiles 22 ms after opening of the RYR1, but these stabilize very rapidly, after a few ms **(G)** Extended colour code with marking of the average amplitude of a microdomain in unstimulated T cells (Diercks et al., 2018) **(H)** Evolution of the amplitude of the simulated Ca^2+^ microdomains with the number of simultaneously open RYR1 in the junction, showing that experimentally observed microdomains do not agree with the opening of the RYRs inside the junction given the low contribution of the opening of the ORAI1 in conditions of a full ER (see text). Dotted line represents junctional Ca^2+^ concentration reached in the absence of ORAI1 channels **(I)** Individual evolution of 1–5 ORAI1 channels open state (Li et al., 2011) as a result of 0–9 RYR1 opening simultaneously. See Supplementary Information and Anim. S1a,b for details.

We simulated the junction schematized in Figure 1 considering an increasing number of open RYR1s during 64 ms. A few milliseconds after RYR1 opening, a stable profile of Ca^2+^ increase in the junction is observed (Figures 2A–F, Supplementary Figure S2). Although *C*_*c*_ can locally reach concentrations close to 20 μM, the average Ca^2+^ concentration in the junction ranges from 340 to 2,500 nM depending on the number of open RYR1 (Anim. S1a,b in the Supplementary Information). Thus, in this configuration, opening of a single RYR1 allows to reach the experimentally observed microdomain amplitude in the junction. To assess the relative contributions of Ca^2+^ entry through ORAI1 and Ca^2+^ release from the ER through RYR1, we performed the same simulations in the absence of ORAI1 in the PM. As visible in Figure 2H in which Ca^2+^ microdomains with and without activated ORAI1 are seen to have nearly the same amplitude, the relative contribution of Ca^2+^ entry is very limited in these conditions. In agreement with this observation, the increase in the amplitude of the Ca^2+^ signal in the microdomain is not related to significant changes in the opening states of ORAI1 (Figure 2I). For example, when three RYR1s open simultaneously, all five ORAI1 channels are still in their lower state of activity as in the absence of any RYR1 opening. These computational observations indicate that the geometry depicted in Figure 1 does not reflect the situation encountered in T-lymphocytes early after TCR activation, since the number of microdomains decreases significantly in ORAI1^−/−^ T cells (Diercks et al., 2018).

### Ca^2+^ Microdomains Observed Soon After T Cell Stimulation Are due to the Opening of Type 1 Ryanodine Receptors Localized on Conic ER Around the ER-PM Junction

**FIGURE 3:**
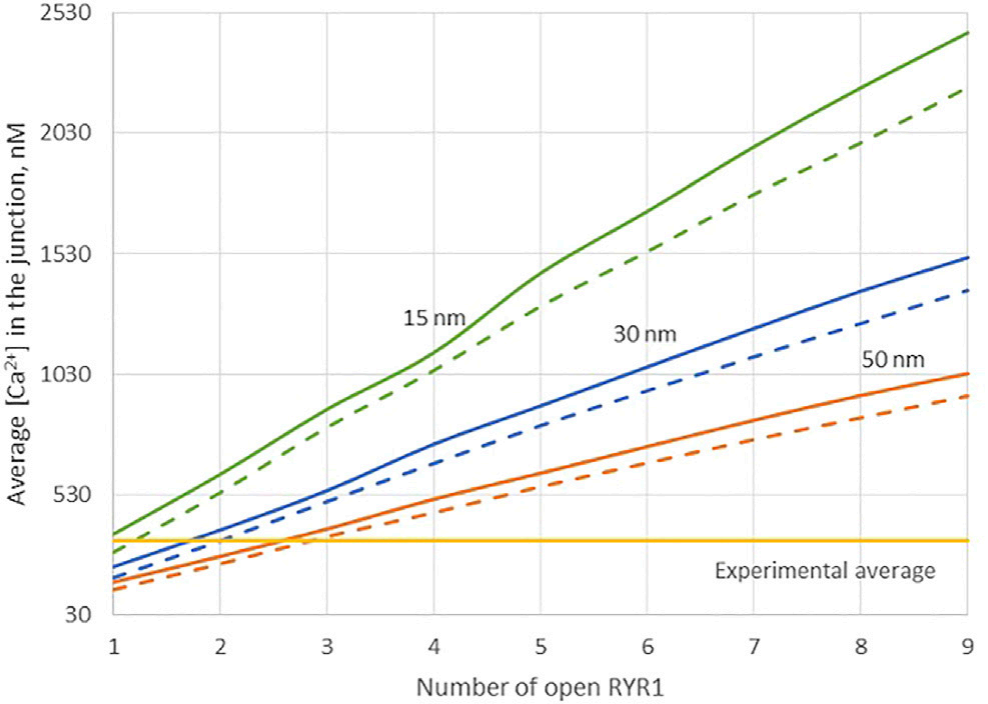
Influence of the value of the distance between the PM and the ERM on the Ca^2+^ microdomains in the ER-PM junction. The green curve (15 nm) corresponds to the situation considered in Figure 2. Larger distances, blue curve (30 nm) and orange curve (50 nm) do not influence the low contribution of the opening of the ORAI1 to the Ca^2+^ concentration increase in the junction. Dotted lines represent junctional Ca^2+^ concentration reached in the absence of ORAI1 channels.

Because of the small size of the junction, actual Ca^2+^ concentrations are expected to be highly sensitive to the ER-PM distance. Thus, results obtained in the previous section may depend on this junctional depth, which led us to investigate the influence of this distance on the Ca^2+^ profile in the junction. Data indicate that the ER-PM spacing is typically 10–20 nm (Hogan, 2015), but we investigated distances up to 50 nm that might be reached locally. As visible in Figure 3, although the amplitude of the Ca^2+^ microdomain is inversely proportional to the height of the junction, the experimental average is reached with less than three RYR1 simultaneously open even for the largest junction considered (50 nm). Moreover, the amplitudes are not much affected by the absence of ORAI1, as visible by the fact that the Ca^2+^ microdomain amplitudes without ORAI1 (dashed lines) are close to those with ORAI1 (plain lines). This does not agree with experimental observations showing that in cells that do not express ORAI1, the frequency of occurrence of junctional microdomains is reduced by ∼10, while their amplitude is lowered by ∼25% (Diercks et al., 2018). We thus concluded that RYR1 located inside the junction, whatever its height, cannot account for experimental observations.

**FIGURE 4:**
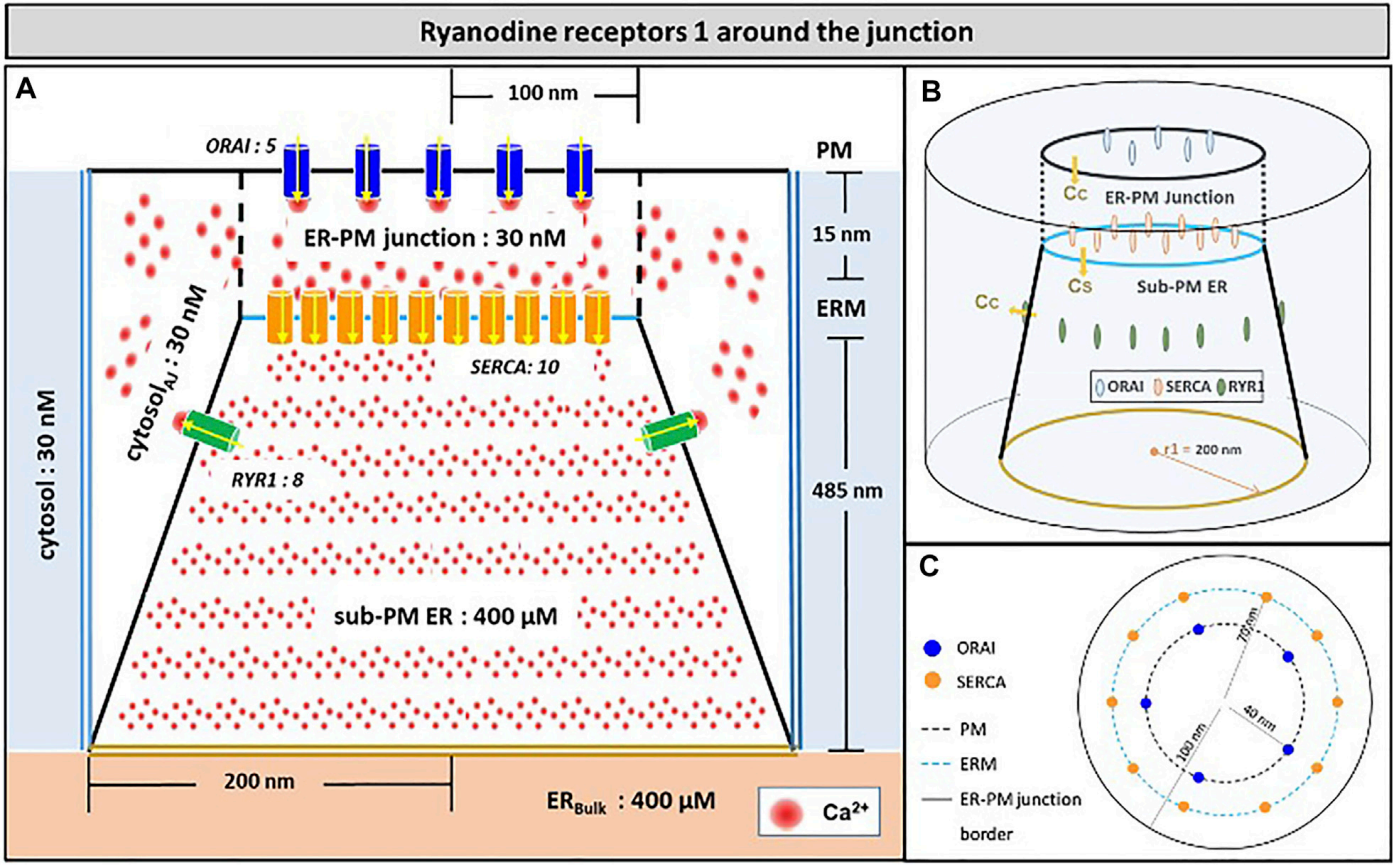
Schematic representation of the model geometry of the ER-PM junction and sub-PM ER used to investigate the origin of the Ca^2+^ microdomains in T cells with eight RYR1 around the junction **(A)** Frontal diagram showing the dimensions of the cone that represents the sub-PM ER, of the junction and of the portion of the cytosol considered in the simulations. ORAI1 channels are in blue, SERCA pumps in orange and RYR1 in green. Plain lines represent membrane boundaries; dashed lines, fictitious limits between the junction and the cytosol and double lines indicate the limits of the simulated system. The resting Ca^2+^ concentrations considered as initial conditions and boundary conditions in the two compartments are indicated. **(B)** 3D view of the model geometry **(C)** Upper view of the positions of the ORAI1 channels on the PM, in blue, and of the SERCA pumps and RYR1 on the ERM, in orange and green respectively. Not to scale. This geometry is based on McIvor et al. (2018). See text for details.

**FIGURE 5:**
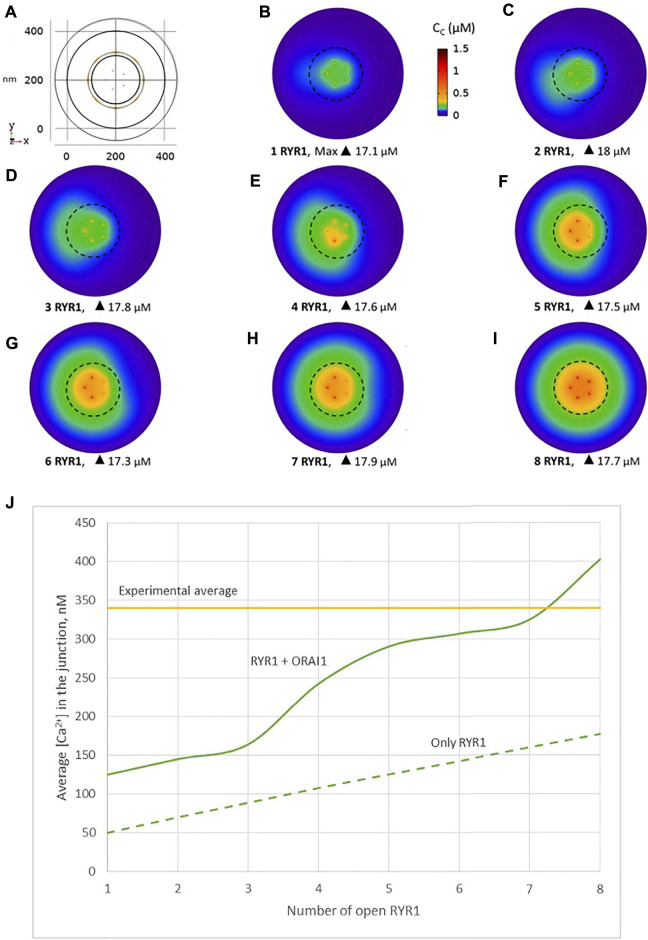
Simulated Ca^2+^ microdomains resulting from the opening of the RYR1 adjacent to the junctions, which in turn induces the opening of ORAI1 channels in the junctions as a result of local depletion of ER Ca^2+^ **(A)** Upper view of the arrangement of the ORAI1 channels on the PM of the junction (red dots) and of the adjacent RYR1 (yellow lines) using COMSOL **(B–I)** Steady-state Ca^2+^ profiles in the junction when opening 1 **(B)** to 8 **(I)** RYR1 simultaneously. Shown are the profiles 22 ms after opening of the RYR1. Upon depletion of local Ca^2+^ in the ER, which is quasi-instantaneous, ORAI1 channels open to an extent that depends on this local concentration, as defined by the function *f*_*2/1*_ (see Supplementary Information). ORAI1 opening is assumed to occur immediately after depletion because ORAI1-STIM1/2 aggregates are pre-formed (Weiss and Diercks, unpublished results). **(J)** Evolution of the amplitude of the simulated Ca^2+^ microdomains with the number of simultaneously open RYR1 in the junction, showing that experimentally observed microdomains can in principle result from the opening of ORAI1 channels induced by the spontaneous opening of a few RYR1 near the junction, in conditions of a full ER. Dotted line represents junctional Ca^2+^ concentration reached in the absence of ORAI1 channels. See Anim. S2a,b,c,d and S3 in Supplementary Information.

Another possibility would be that RYR1s are located outside the junction, but close to it, in such a way that they affect sub-PM ER Ca^2+^ concentration. Thillaiappan et al. (2017) reported clusters of immobile IP_3_Rs surrounding the ER-PM junctions, with the mouths of the IP_3_Rs directed towards the PM. In our previous computational study of the IP_3_R-dependent, adhesion-induced Ca^2+^ microdomains, we found that simulations based on this configuration agree with experimental observations (Gil et al., 2021). We investigated the possibility that RYR1s are similarly localized around the junction. In this configuration, schematized in Figure 4, a ring of RYR1s located in the sub-PM ER membrane and spaced by 90 nm, are releasing Ca^2+^ in the cytosolic space adjacent to the junction. The Ca^2+^ microdomains simulated under this configuration are shown in Figure 5, considering 1 (Figure 5B, Supplementary Figure S2) to 8 (Figure 5I) open RYR1. To reach the experimentally observed average amplitude of around 340 nM, seven or eight RYR1 must open simultaneously (Figure 5J). This number is slightly affected by the distance between the RYR1 and the junction. If a 45 nm distance is considered, instead of 90 nm as considered in Figure 5, opening of five RYR1s simultaneously is sufficient to reach the experimentally observed amplitude in the junction (Supplementary Figure S4). Indeed, a larger amount of the Ca^2+^ released by RYR1 can diffuse into the junction in this configuration. In the absence of ORAI1, the increase in Ca^2+^ in the junction due to RYR1 opening is much below the experimentally observed amplitude of the microdomains. Thus, when the RYR1 located in the membrane of the sub-PM ER are not releasing Ca^2+^ directly in the junctions, the model reproduces the experimental observation that NAADP-induced Ca^2+^ microdomains rely on both RYR1 and ORAI1 (Diercks et al., 2018).

**FIGURE 6:**
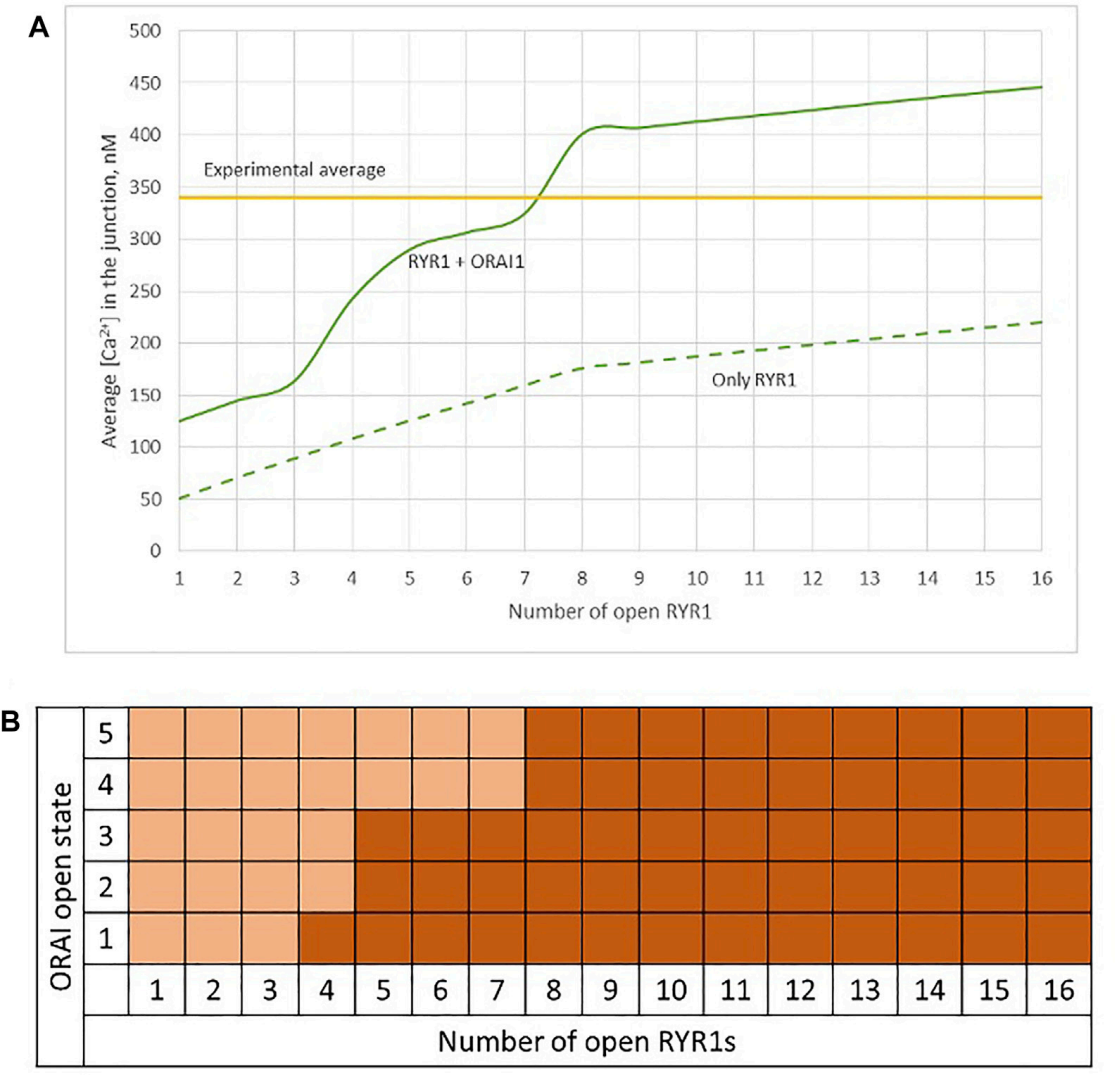
Simulated Ca^2+^ microdomains resulting from the opening of up to 16 RYR1 adjacent to the junctions **(A)** Evolution of the amplitude of the simulated Ca^2+^ microdomains in the presence (green curve) and in the absence of ORAI1s (dotted green curve) in the junction. Both green curves, up to eight simultaneously open RYR1, correspond to the situation considered in Figure 5. The theoretical situation of a junction that does not contain ORAI1 channels (dotted green curve) allows to appreciate that the contribution of Ca^2+^ released through the RYR1 to the Ca^2+^ microdomain is linear and rather limited. At eight simultaneously open RYR1 (green curve), the complete cluster of five ORAI1 channels reach their maximum open state possible, in conditions of a full ER **(B)** Individual evolution of 1–5 ORAI1 channels open state (Li et al., 2011) as a result of 1–16 RYR1 opening simultaneously. See Supplementary Information for details.

**FIGURE 7:**
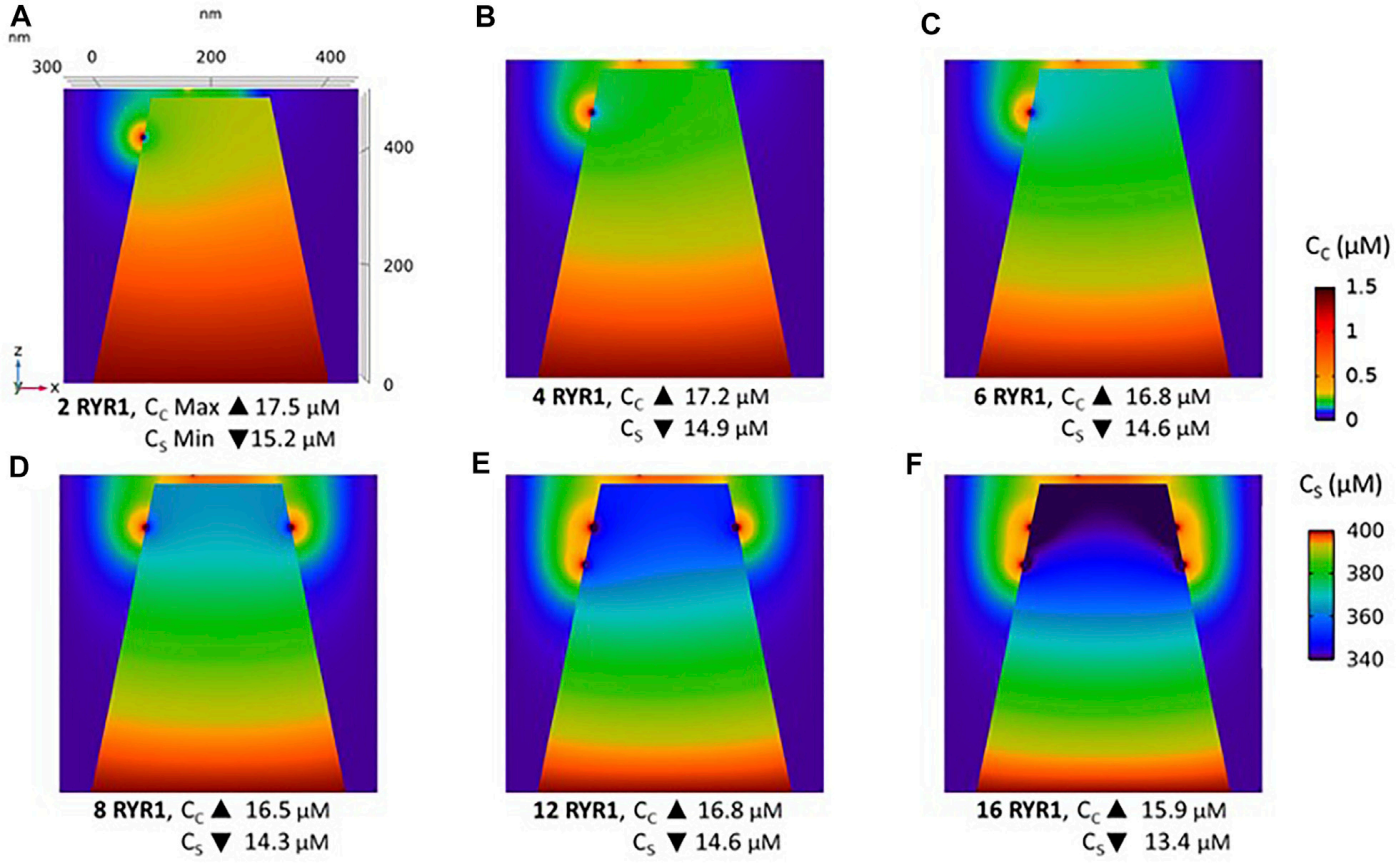
Cross-section of the Ca^2+^ profiles in the junction, in the cytosol adjacent to the junction and in the sub-PM ER during microdomain formation **(A–D)** microdomains created by the opening of 2, 4, 6 and 8 RYR1. Local depletion of ER Ca^2+^ provokes the opening of the nearby ORAI1s. This situation corresponds to the one shown in Figure 5. **(E,F)** microdomains created by the opening of 12 and 16 RYR1, respectively. The second cluster of eight RYR1 is located directly underneath the first cluster. Local depletion of ER Ca^2+^ is not enough to provoke additional opening of the nearby ORAI1s. This situation corresponds to the one shown in Figure 6. For all panels, the upper right bar indicates the colour code of Ca^2+^ concentration in the cytosol while the lower right bar indicates the colour code of Ca^2+^ concentration in the ER. See Anim. S4.

Observations in T cells indicate that Ca^2+^ signals in the junction are rather stereotypic, with a relatively constant amplitude (Diercks et al., 2018). It is thus expected that the microdomain characteristics are not very sensitive to the numbers of RYR1 present around the junction. We next investigated the influence of the number of open RYR1 in more detail, considering the possibility that up to 16 RYR1 are located around the junction. This was done in the simulations by considering another ring of eight receptors 90 nm below the first one. As visible in Figure 6A, the relation between the amplitude of the simulated microdomains and the number of open RYR1 is non-linear with a marked stepwise behaviour. From one to three open RYR1, the increase in amplitude is linear. All five STIM1/2 bound ORAI1 channels are in their lowest conductance state (Figure 6B) and the Ca^2+^ increase in the junction is due to diffusion from the adjacent cytosol. From four open RYR1 on, local ER Ca^2+^ depletion is sufficient to further activate ORAI1 creating changes in the slope of the relation between the amplitude of the Ca^2+^ signal and the number of open receptors. If more than eight RYR1 open simultaneously, additional ones do not activate ORAI1 further. As seen in Supplementary Figure S1, the 54% opening state is reached when the value of *C*_*S*_ in the close vicinity of STIM1/2 bound to ORAI1 reaches 260 μM. This would require a Ca^2+^ decrease at the ER lumen close to RYR1 channel that is not reached under localised Ca^2+^ signaling because of fast diffusion-mediated replenishment. From 8 to 16 open RYR1, local depletion is not much affected, with a minimal ER Ca^2+^ concentration that remains around 340 μM as visible in Figure 7 that shows a cross-sectional view of the Ca^2+^ concentrations in the sub-PM ER and in the cytoplasmic space including the junction (see also Supplementary Figure S3 for cross-sections). Noticeably, Ca^2+^ concentrations at the cytosolic side of RYR1 slightly decrease with the number of open receptors, from 17.5 to 15.9 μM for 2 and 16 open receptors, respectively. The slight decrease in the Ca^2+^ gradient at the channel pore indeed reduces the flux through the RYR1. Thus, increasing the number of RYR1 leads to a decrease in the ER Ca^2+^ concentration around STIM1/2, but this decrease is not sufficient to provoke the passage of ORAI1 channels to a higher conductance state. NAADP-induced Ca^2+^ microdomains observed early after T cell stimulation thus rely on the simultaneous opening of an average of seven–eight RYR1 located around the ER-PM junction.

### Predicted Type 1 Ryanodine Receptors Involvement in the Formation of Microdomains Agree With Type 1 Ryanodine Receptors Open Probabilities in the Presence of NAADP

Simulation results obtained in the previous section indicate that best agreement between modelled and experimentally observed microdomains occur when most of the eight RYR1 located near the preformed ORAI1/STIM1/STIM2 complexes are open simultaneously during 64 ms. This conclusion stems from a direct comparison between the simulated and experimentally observed Ca^2+^ signals. To further validate this result, some reasoning based on RYR1 open probability can be proposed. The 64 ms duration of a Ca^2+^ microdomain corresponds to the average duration of the NAADP-evoked Ca^2+^ signals arising in the first 15 s after TCR activation (Diercks et al., 2018). Interestingly, 64 ms also fits in the range of the reported durations of Ca^2+^ sparks (Jaggar et al., 2000). It is thus likely that in response to the TCR/CD3 stimulation-induced NAADP increase, RYR1 undergo repetitive openings maintained by Ca^2+^-induced Ca^2+^-release, a process that generates a small amplitude Ca^2+^ increase in the cytosol, called “spark”. The decrease of ER Ca^2+^ that accompanies the spark is in turn responsible for the opening of ORAI1, and thus for the Ca^2+^ microdomain in the junction.

**FIGURE 8:**
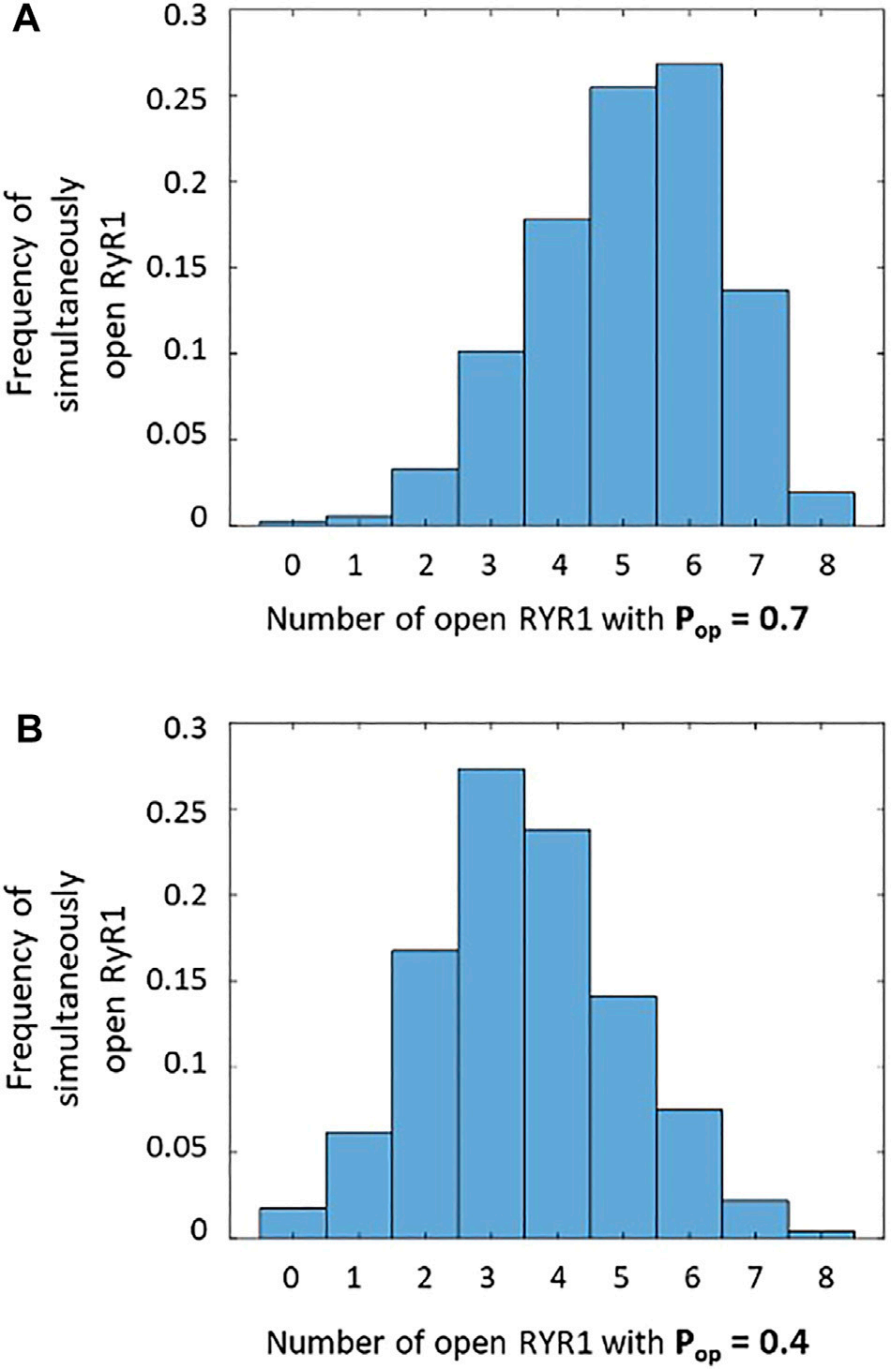
Frequency of simultaneously open receptors in a cluster of eight RYR1 during 64 ms that corresponds to the duration of a Ca^2+^ spark **(A)** During a spark when RYR1 are maximally activated by NAADP, there are most of the time around five to six simultaneously open RYR1, with a single receptor open probability of 0.7 (Hohenegger et al., 2002). This is in agreement with the results seen in Figure 5. **(B)** At basal NAADP concentration, the single receptor open probability is around 0.4, and there are continuously around three to four simultaneously open RYR1, which is not enough to reach the experimental Ca^2+^ amplitude. In the two panels, the stochastic simulations of opening and closing of individual receptors are performed during 64 ms.

We thus investigated if our conclusions about the molecular mechanism underlying TCR/CD3-induced Ca^2+^ microdomains are compatible with the dynamics of RYR1 during spark-like activity. Upon TCR/CD3 stimulation, global NAADP concentration in T cells increases from 4.1 ± 1.5 nM to 33.6 ± 7.2 nM (Gasser et al., 2006). Because RYR1 are activated by NAADP in T cells (Wolf et al., 2015; Diercks et al., 2018; Roggenkamp et al., 2021), their Ca^2+^-releasing activity will increase. Indeed, Hohenegger et al. (2002) have shown that the open probability of these receptors is a highly nonlinear function of NAADP concentration, with an EC_50_ of 31.2 ± 6.9 nM. Because NAADP synthesis occurs near the ER-PM junctions (Gu et al., 2021), local concentrations in the vicinity of RYR1 are certainly larger than the average values mentioned above and likely exceed the EC_50_. Thus, the open probability of RYR1 near the junctions must be of the order of 0.7, which is the maximal value measured at 20 μM Ca^2+^. Given that the mean open time of RYR1 is ∼2 ms (des Georges et al., 2016; Sato & Bers, 2011), the mean closed time in these conditions can be estimated to be 0.86 ms. On the basis of these data, one can obtain a rough approximation of the number of simultaneously open receptors in a spark site when RYR1s are maximally activated by NAADP. Straightforward stochastic simulations of opening and closing of eight RYR1 with average opening and closing times equal to 2 and 0.86 ms, respectively, indicate that most of the time, six receptors are simultaneously open (Figure 8A). This result was obtained by performing 64 ms long stochastic simulations of eight independent RYR1 and determining at each time step how many receptors are open. The maximal frequency at six open receptors is in accordance with the three-dimensional spatio-temporal simulations indicating that best agreement between experimental observations and computational results is obtained when seven to eight RYR1 are simultaneously open during a 64 ms Ca^2+^ spark, considering the existence of pre-formed STIM1/2 and ORAI1 complexes (Figure 5). In contrast, the same calculations predict that for basal NAADP concentrations, when the open probability of RYR1 equals 0.4, the highest frequency corresponds to three RYR1 open simultaneously (Figure 8B). In the above simulations (Figure 5), this corresponds to a Ca^2+^ microdomain with an amplitude well below the experimental average. Similar stochastic simulations (Supplementary Figure S5 and related text in the SI) indicate that for the IP_3_-mediated microdomains corresponding to pre-stimulation conditions, the maximal frequency corresponds to two receptors open simultaneously, in qualitative agreement with our previous results (Gil et al., 2021).

In summary, by combining previous observations about NAADP increase upon TCR/CD3 stimulation of T cells and RYR1 open probability, we conclude that the NAADP-induced increase in the open probability of RYR1 triggers the opening of most of the eight RYR1 located around the ER-PM junction. This is in agreement with the spatio-temporal simulations shown in the previous section, which indicate that Ca^2+^ microdomains observed early after T cell stimulations rely on the simultaneous opening of seven to eight RYR1 located around the junction.

### The Isoforms of STIM That Are Bound to ORAI1 Determine the Characteristics of the Ca^2+^ Microdomains

In the previous sections, we found that the microdomains occurring during the first 15 s after TCR/CD3 stimulation involve a larger number of ER Ca^2+^ releasing channels than those observed before stimulation. Indeed, the local depletion induced by the opening of six–seven RYR1 is needed to activate ORAI1 and reproduce experimentally observed NAADP-dependent microdomains (Figure 5) while three to six IP_3_R are involved in the creation of the adhesion-mediated, IP_3_-dependent microdomains (Gil et al., 2021). At first sight, this is paradoxical as the conductance of RYR1 is about 5 times larger than that of IP_3_Rs.

**FIGURE 9:**
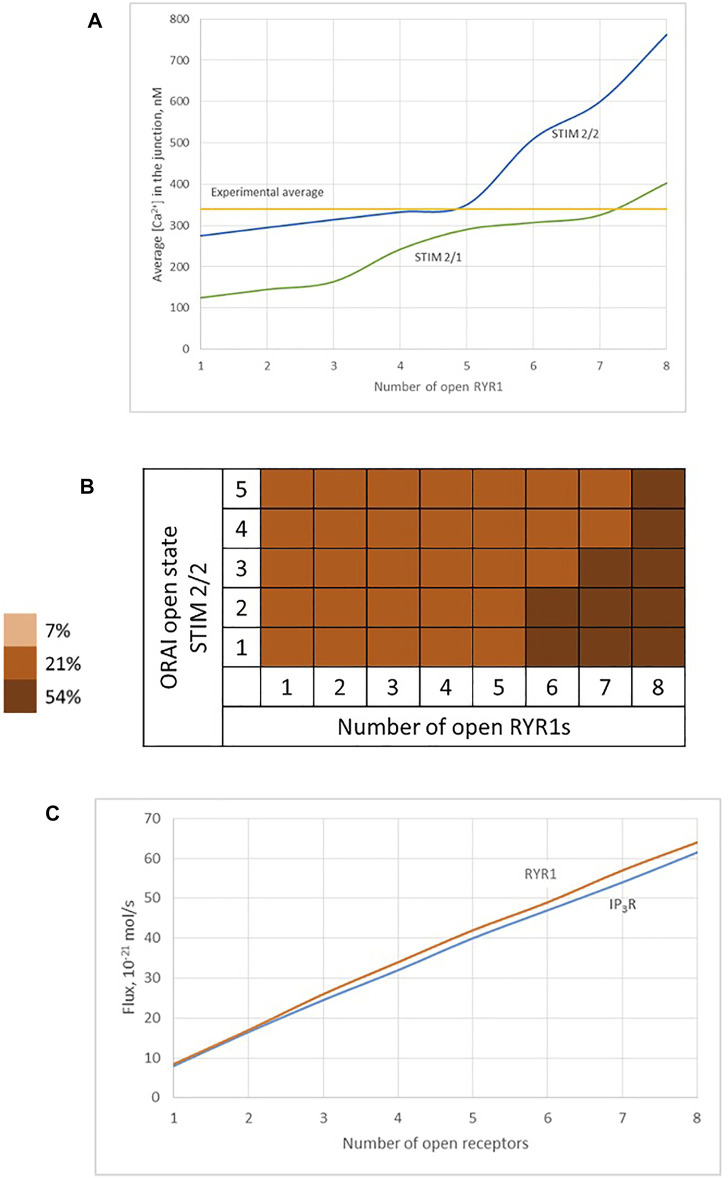
Influence of the nature of the ER Ca^2+^ channel inducing the local depletion of ER Ca^2+^ (IP_3_R or RYR1) and of the STIM isoforms bound to ORAI1 on the characteristics of Ca^2+^ microdomains **(A)** Evolution of Ca^2+^ microdomains amplitude with the number of simultaneously open RYR1 in the junction. The microdomains observed in conditions of full ER can be induced by the spontaneous opening of a few RYR1 near the junction that in turn trigger the opening of ORAI1 channels bound to STIM2/2 (blue curve) or to STIM1/2 (green curve). ORAI1 channels open to an extent that depends on local ER Ca^2+^ concentration, as defined by the corresponding function *f*_*2*_ or *f*_*2/1*_ (see Supplementary Information) **(B)** Individual evolution between the 5 open states of the ORAI1 (Li et al., 2011), as a result of 1–8 RYR1 opening simultaneously. **(C)** Comparison of Ca^2+^ fluxes through open IP_3_Rs and RYR1. Because of the slow replenishment around the pore of the receptor channel with D_S_ = 10 μm^2^/s, the concentration gradient around the two extremities of the pore does not changes drastically and hence the flux remains of the same order for IP_3_Rs and RYR1.

We thus studied the characteristics of the Ca^2+^ increases created by the simultaneous opening of either eight IP_3_Rs or eight RYR1 (see Table 1). For the two Ca^2+^ channel types, we considered two possible preformed ORAI1/STIM complexes: STIM2 homotetramers (STIM2/2) and STIM1 and STIM2 heterotetramers (STIM1/2). Simulation results indicate that the nature of the ER Ca^2+^ release channel does not much influence the characteristics of the Ca^2+^ microdomains. Indeed, the Ca^2+^ signal in the junction is determined by the opening state of ORAI1, which is the same when Ca^2+^ release from the ER is mediated by IP_3_Rs or RYR1. The Ca^2+^ concentration sensed by the ORAI1/STIM complex is nearly the same in the two situations. As shown in Figure 9C, once the steady state gradients are established, the fluxes are nearly identical. Because of the slow replenishment around the pore of the receptor channel with D_S_ = 10 μm^2^/s, the concentration gradient around the two extremities of the pore does not changes drastically and hence the flux remains of the same order for IP_3_R and RYR1. In contrast, the nature of the STIM isoforms bound to ORAI1 has a drastic influence on the characteristics of the Ca^2+^ microdomain since it determines their Ca^2+^ sensitivity, and hence the opening state of ORAI1. Because STIM1/2 has a lower sensitivity to ER Ca^2+^ depletion than STIM2/2, the open state of ORAI1 is lower and the increase in Ca^2+^ in the junction has both a smaller amplitude and spatial extent (compare blue to green lines in Figure 9A,B to Figure 6B and Table 1).

**TABLE 1:** Characteristics of the simulated microdomains relying on the simultaneous opening of eight IP_3_Rs for 44 ms (first two lines) or of eight RYR1 for 64 ms (lines three and 4). For each case, two situations are considered: the existence of pre-formed clusters of ORAI1 with STIM2/2 homotetramers (lines 1 and 3) and the existence of pre-formed clusters of ORAI1 with STIM2/1 heterotetramers (lines two and 4). Maximal concentrations denote the maximal local concentrations reached in the domains indicated. Spatial extent refers to the area of the junction’s portion in which Ca^2+^ concentration exceeds 300 μM. The ER [Ca^2+^] felt by ORAI is the average local concentration of luminal Ca^2+^ around the mouth of the IP_3_R or RYR1, computed in a 108 nm^3^ volume.

Eight Open Receptors		Max[Ca^2+^] in the junction(μM)	Max[Ca^2+^] around the junction(μM)	Average[Ca^2+^] in the junction(μM)	Spatial extent (μM^2^)	ER[Ca^2+^] at channel pore(μM)	ER[Ca^2+^] felt by ORAI(μM)	ORAI mean open state
IP_3_R 44 ms	STIM2/2	10	17	0.755	0.049	11	331	54%
	STIM2/1	354	17	0.396	0.024	11	331	21%
RyR 165 ms	STIM2/2	11	18	0.762	0.049	12	328	54%
	STIM2/1	4.57	18	0.402	0.024	12	328	21%

**FIGURE 10:**
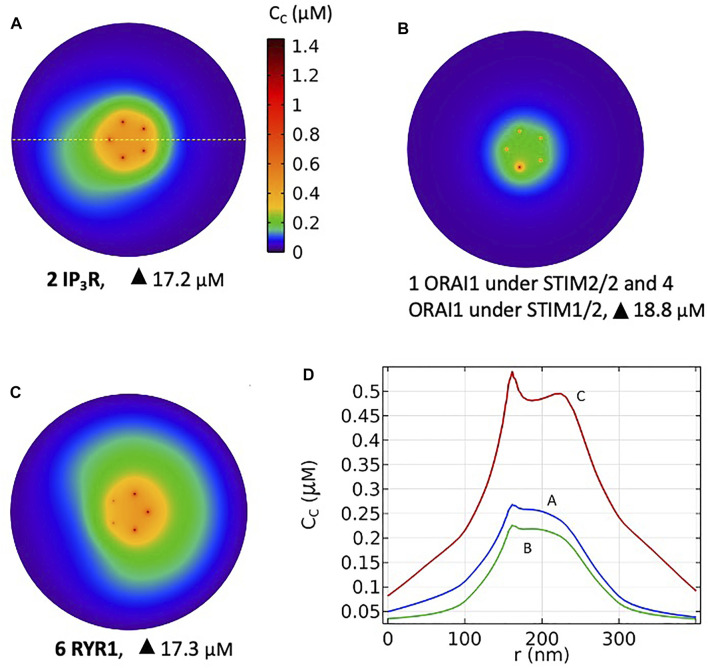
Simulated most probable Ca^2+^ microdomains resulting from a T cell transition between quiescent to early activation. **(A)** Non TCR/CD3-dependent Ca^2+^ microdomains formed by the opening of two IP_3_Rs adjacent to the junction and further opening of ORAI1 channels bound to STIM2/2 **(B)** Basal opening of five ORAI1 channels, one inherently co-localized with STIM2/2 and four inherently co-localized with STIM1/2 leading to small microdomains arising from nano-scale [Ca^2+^] fluctuations in the sub-PM ER. Artificial construction **(C)** TCR/CD3-dependent Ca^2+^ microdomains formed by the opening of six RYR1 adjacent to the junction and further opening of ORAI1 channels bound to STIM1/2. See Anim. S5a,b,c in the Supplementary Information. **(D)** Ca^2+^ profiles along the yellow dotted line shown in A that traverses the middle of the junction at a 7.5 nm distance from the PM, corresponding to panels A, B and C.

Based on these computational observations, the prototypical evolution of Ca^2+^ microdomains from adherent to TCR/CD3 stimulated T cells is proposed to obey the following scenario. Upon adhesion to proteins of the extracellular matrix, integrin evoked IP_3_ signaling provokes an increase in the frequency of Ca^2+^ puffs arising from the clusters of IP_3_R located near the ER/PM junction. These puffs typically last ∼44 ms during which, in average, two IP_3_Rs are open simultaneously. On the other hand, five ORAI1 channels are located in the PM of the junction and bound to STIM2/2 homotetramers. In response to the decrease in ER Ca^2+^ created by the puff, the ORAI1 channels open to ∼21% of their maximal conductance and create the Ca^2+^ microdomain (Figures 10A, 1^st^ row of Table 2). After the Ca^2+^ puff, Ca^2+^ is rapidly replenished in the sub-PM ER and ORAI1 channels shift to their lowest conductance state (∼7% opening) that corresponds to basal Ca^2+^ entry, but not to a detectable Ca^2+^ microdomain (Figure 10B, 2nd row of Table 2) when bound to the STIM1/2 isoform, and stay at 21% when bound to STIM2/2. TCR/CD3 stimulation initiates NAADP signaling, which increases the open probability of the RYR1 located around the junction and thus the frequency of Ca^2+^ sparks (Figure 10C, 3rd row of Table 2). During these events, ∼6 RYR1 are open simultaneously. At this stage, ORAI1 channels are preferably bound to STIM1/2 heterotetramers, which decreases their sensitivity to ER Ca^2+^ depletion. Thus, as in the case of the IP_3_-dependent microdomains, they open at ∼21% of their full capacity. However, because more Ca^2+^ is released in the cytosolic space just around the junction by six RYR1 than by two IP_3_R, the Ca^2+^ microdomain in the junction is a bit larger because of diffusion.

**TABLE 2:** Characteristics of the simulated microdomains corresponding to IP_3_-dependent Ca^2+^ signaling stimulated by T cell adhesion (line 1), to a spontaneous opening of ORAI in the absence of stimulation (line 2) or to NAADP-dependent Ca^2+^ signaling in response to TCR/CD3 stimulation (line 3)

—	Average[Ca^2+^] in the junction(μM)	Spatial extent (μm^2^)	ER[Ca^2+^] felt by ORAI(μM)	ORAI mean open state
Puff,2 IP_3_R; STIM2/2	0.293	0.0078	350	21%
ORAI,STIM2/2 to STIM2/1	0.236	—	400	7%
Spark,6 RYR1; STIM2/1	0.307	0.0123	335	21%

## Discussion

**FIGURE 11:**
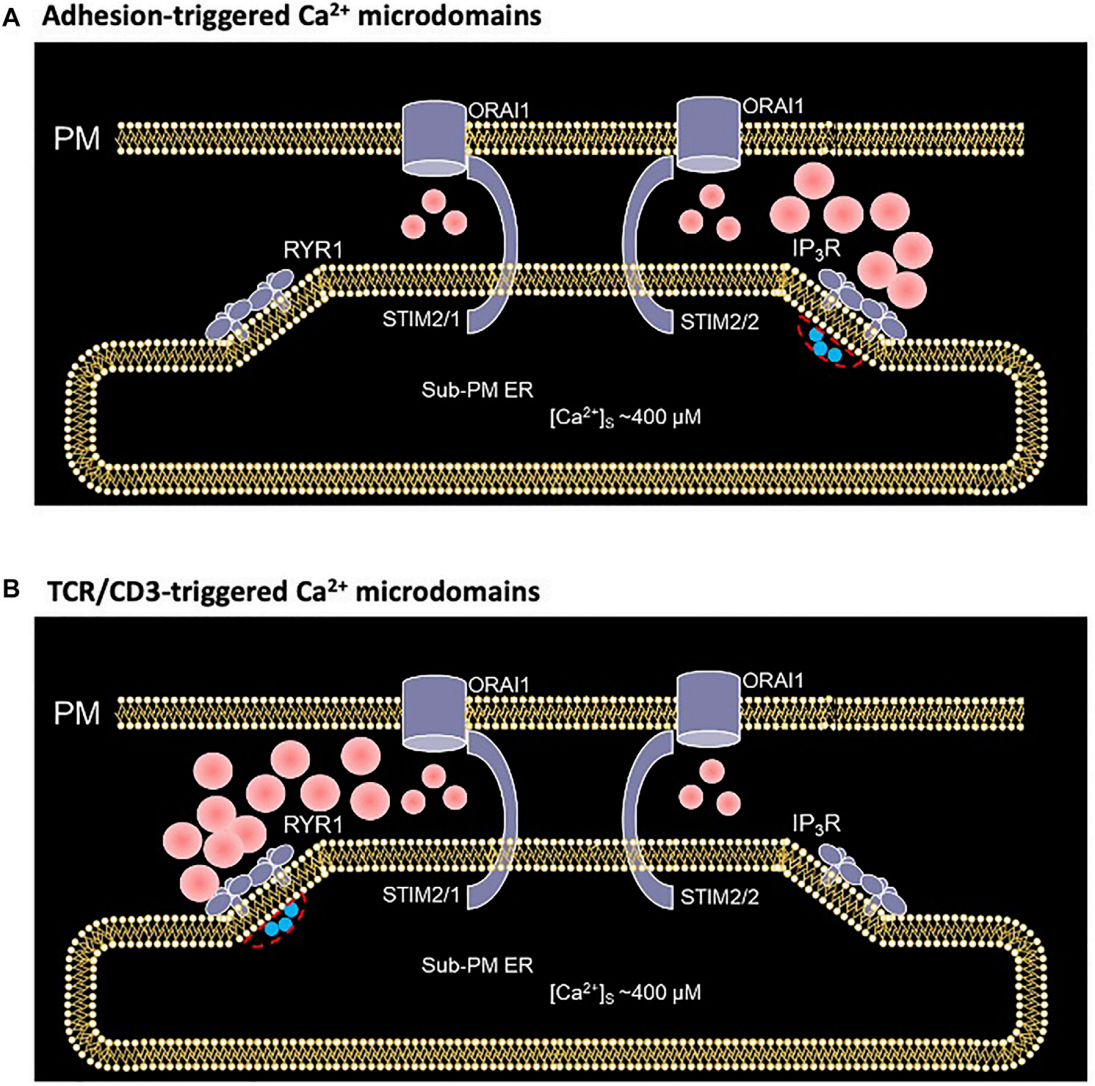
Schematized representation of the proposed mechanism underlying the spontaneous formation of Ca^2+^ microdomains in T cells during its transition from quiescent to early activation **(A)** In an otherwise unstimulated cell, non TCR/CD3 dependent short, spontaneous activation of one or a few IP_3_Rs close to the junction, releases Ca^2+^ from the sub-PM ER into the cytosol, leading to further opening of ORAI1 channels most likely bound to STIM2/2 at this stage (Weiss and Diercks, unpublished results) **(B)** During the first 15 s following TCR/CD3 stimulation, and NAADP driven activation of several RYR1 close to the junction, slightly larger amount of Ca^2+^ is released from the sub-PM ER into the cytosol. The resulting local Ca^2+^ depletion close to the RYR1 pore provokes the unbinding of Ca^2+^ from STIM1/2 heterotetramers, which further activates ORAI1 channels (Diercks et al., 2018). Red spots represent Ca^2+^ ions.

Activation of T cells is an essential step to start an adaptive immune response. At this particular point a highly important decision is made: whether a T cell stays quiescent or may develop into an effector T cell carrying out immune effector functions to destroy pathogens, or in case of autoimmune reactions, to attack our own body. Among several signaling processes involved, Ca^2+^ signaling is fundamental for T cell activation. In a previous study, we resorted to mathematical modeling to gain insight into the early, small scale Ca^2+^ increases that follow adhesive interactions of T cells, which play a crucial role in T cell migration to inflamed tissue (Weiss and Diercks, unpublished results; Mezu-Ndubuisi & Maheshwari, 2021). Here, we extend this model to investigate the molecular mechanism underlying the early phase of activation following TCR/CD3 stimulation. Although both types of Ca^2+^ microdomains largely rely on Ca^2+^ entry through preformed ORAI1/STIM complexes, the former response relies on IP_3_ signaling while the latter involves NAADP and RYRs. Interestingly, a progressive change in STIM isoforms, from mainly STIM2 homotetramers to STIM1/2 heterotetramers, also accompanies this transition (Figure 11). Computational modeling of the spatio-temporal Ca^2+^ dynamics in the ER-PM junctions allowed us to reproduce the observation that the microdomains triggered by cell adhesion or by TCR/CD3 stimulation at its early phase appear rather similar, despite the different underlying mechanisms.

Simulations of the NAADP dependent microdomains quite forwardly predict that RYR1 are most probably located outside the junction, in a region of the ER membrane that is directly adjacent to the junction. This view contrasts with the well-known arrangement of RYR2 in cardiac cells, where they are facing the PM in dyadic clefts (Jones et al., 2018). In principle, a few RYR1 could be located in the ER-PM junction of T cells because the dimensions of its cytoplasmic part (approx. 28 nm × 28 nm × 12 nm, see Lanner et al., 2010) do not exceed the dimensions of the junction. However, Ca^2+^ microdomains simulated with such a spatial arrangement are no longer dependent on ORAI1, since the increase in Ca^2+^ concentration due to the influx mediated by a single RYR into the junction suffices to create a Ca^2+^ signal of the amplitude observed experimentally, which does not agree with experimental results. It is thus most probable that in T cells RYR1 are arranged around the junction, in the same way as IP_3_R (Thillaiappan et al., 2017; Taylor and Machaca, 2019).

Computational results indicate that the influx through ORAI1 channels much depends on the STIM isoforms to which it is bound. In the case of local signaling investigated here, the same opening state of ORAI1 is reached after local Ca^2+^ depletion induced by three IP_3_R when it is bound to STIM2/2 as after local Ca^2+^ depletion induced by seven RYR1 when it is bound to STIM1/2. Thus, the change in the nature of the ORAI1/STIM complexes that follow TCR/CD3 stimulation is expected to play a crucial role in maintaining Ca^2+^ signaling localized despite the stimulation of RYR by NAADP. Along this line, Ahmad et al. (2021) have recently shown in HEK293 cells that while clusters of STIM2 represent the sites of SOCE initiation, STIM1 molecules are progressively recruited when cells are exposed to low stimulation.

In contrast, the nature of the ER-releasing Ca^2+^ channel that creates the local depletion in the sub-PM ER, subsequently triggering ORAI1 opening does not have a significant effect. This result is *a priori* surprising given that the RYR1 has a conductance ∼5 times larger than the IP_3_R. Simulations indicate that the flux is limited by the replenishment of ER Ca^2+^ at the mouth of the channel rather than by its conductance. Thus, the extent of local depletion is imposed by the value of the diffusion coefficient of Ca^2+^ in the ER. This computational observation agrees with the major role played by intraluminal diffusion of Ca^2+^ in setting the responsiveness of Purkinje cells to synaptic inputs (Okubo et al., 2015). It should be kept in mind that the peculiar geometry of the junctional ER is expected to play an important role in decreasing the value of the effective Ca^2+^ diffusion coefficient because of the tortuosity of the tubular network of the ER (Schaff et al., 1997; Oloveczky and Verkman, 1998). In our simulations, diffusion is however fast enough to avoid decreases in local ER Ca^2+^ that would trigger the passage of ORAI1 channels in a highly active state. Simulations indicate that during localized Ca^2+^ signaling in T cells, ORAI1 channels never exceed 21% of their maximal activity.

Together with experimental observations (Diercks et al., 2018; Weiss and Diercks, unpublished results), our computational model ascribes the evolution of Ca^2+^ microdomains from the adherent/pre-stimulated to the TCR/CD3 early stimulated state as a passage from puff-to spark-triggered SOCE. Indeed, while the two types of localized Ca^2+^ signaling rely on ORAI1-mediated Ca^2+^ entry, cell adhesion triggers the synthesis of IP_3_, and TCR/CD3 stimulation initially that of NAADP. The respective durations of the two types of Ca^2+^ microdomains (44 ± 4 ms and 64 ± 3 ms) are in the ranges of those reported for puffs (Bootman et al., 1997) and sparks (Jaggar et al., 2000), respectively. In the two cases, the local depletion in ER Ca^2+^ created by the puff or the spark can trigger ORAI1 opening, with a resulting simulated Ca^2+^ increase in the ER-PM junction that matches experimental observations. Moreover, straightforward stochastic simulations of channel opening and closing taking into account the channels open probabilities in the presence of ligand and high Ca^2+^ concentration indicate a number of simultaneously open receptors matching with the results of the 3D spatiotemporal simulations. In the future, more realistic 3D simulations should be performed to take into account the stochastic nature of puffs and sparks to simulate microdomains, instead of the simplified deterministic description of stereotypic IP_3_R- or RYR1-mediated release of ER Ca^2+^ used in the present study.

As another perspective, the present model could be used to investigate how the increase in frequency of RYR1 opening observed ∼15 s after TCR/CD3 stimulation affects the spatiotemporal dynamics of junctional Ca^2+^ during the transition of T cells towards full activation. In these longer time scales, additional aspects of SOCE regulation should be considered, such as slow Ca^2+^ dependent inactivation (Dagan and Palty, 2021) or the dynamic nature of the ER-PM junctions (Okeke et al., 2016). The extension of the model to several junctions would enable to investigate how microdomains spread and interact to propagate Ca^2+^ signals deeper into the cell and promote full activation.

## Data Availability

The original contributions presented in the study are included in the article/Supplementary Material, further inquiries can be directed to the corresponding author.

## References

[B1] Ahmad M. Ong H. L. Saadi H. Son G. Y. Shokatian Z. Terry L. E. (2021). Functional Communication between IP_3_R and STIM2 at Sub-threshold Stimuli Is a Critical Checkpoint for Initiation of SOCE. Proc. Natl. Acad. Sci. U S A 119 (3), e2114928118. 10.1073/pnas.2114928118 PMC8784118 35022238

[B3] Bootman M. Niggli E. Berridge M. Lipp P. (1997). Imaging the Hierarchical Ca2+ Signalling System in HeLa Cells. J. Physiol. 499, 307–314. 10.1113/jphysiol.1997.sp021928 9080361 PMC1159306

[B4] Brandman O. Liou J. Park W. S. Meyer T. (2007). STIM2 Is a Feedback Regulator that Stabilizes Basal Cytosolic and Endoplasmic Reticulum Ca2+ Levels. Cell 131 (7), 1327–1339. 10.1016/j.cell.2007.11.039 18160041 PMC2680164

[B5] Dagan I. Palty R. (2021). Regulation of Store-Operated Ca2+ Entry by SARAF. Cells 10, 1887. 10.3390/cells10081887 34440656 PMC8391525

[B7] des Georges A. Clarke O. B. Zalk R. Yuan Q. Condon K. J. Grassucci R. A. (2016). Structural Basis for Gating and Activation of RyR1. Cell 167 (1), 145–157. 10.1016/j.cell.2016.08.075 27662087 PMC5142848

[B8] Diercks B. P. Werner R. Weidemüller P. Czarniak F. Hernandez L. Lehmann C. (2018). ORAI1, STIM1/2, and RYR1 Shape Subsecond Ca2+ Microdomains upon T Cell Activation. Sci. Signal. 11 (561), eaat0358. 10.1126/scisignal.aat0358 30563862 PMC6728084

[B9] Feske S. (2007). Calcium Signalling in Lymphocyte Activation and Disease. Nat. Rev. Immunol. 7, 690–702. 10.1038/nri2152 17703229

[B10] Gasser A. Bruhn S. Guse A. H. (2006). Second Messenger Function of Nicotinic Acid Adenine Dinucleotide Phosphate Revealed by an Improved Enzymatic Cycling Assay. J. Biol. Chem. 281 (25), 16906–16913. 10.1074/jbc.m601347200 16627475

[B11] Gil D. Guse A. H. Dupont G. (2021). Three-Dimensional Model of Sub-plasmalemmal Ca2+ Microdomains Evoked by the Interplay between ORAI1 and InsP3 Receptors. Front. Immunol. 12, 659790. 10.3389/fimmu.2021.659790 33995380 PMC8113648

[B12] Gu F. Krüger A. Roggenkamp H. Alpers R. Lodygin D. Jaquet V. (2021). DUOX2 Synthesizes NAADP in the Early Phase of T Cell Activation. Sci. signaling 14, eabe3800. 10.1126/scisignal.abe3800 34784249

[B13] Guo T. Gillespie D. Fill M. (2012). Ryanodine Receptor Current Amplitude Controls Ca 2+ Sparks in Cardiac Muscle. Circ. Res. 111 (1), 28–36. 10.1161/circresaha.112.265652 22628577 PMC3417769

[B14] Guse A. H. Gil Montoya D. C. Diercks B.-P. (2021). Mechanisms and Functions of Calcium Microdomains Produced by ORAI Channels, D-Myo-Inositol 1,4,5-trisphosphate Receptors, or Ryanodine Receptors. Pharmacol. Ther. 223, 107804. 10.1016/j.pharmthera.2021.107804 33465399

[B15] Hogan P. G. (2015). The STIM1-ORAI1 Microdomain. Cell calcium 58 (4), 357–367. 10.1016/j.ceca.2015.07.001 26215475 PMC4564343

[B16] Hohenegger M. Suko J. Gscheidlinger R. Drobny H. Zidar A. (2002). Nicotinic Acid-Adenine Dinucleotide Phosphate Activates the Skeletal Muscle Ryanodine Receptor. Biochem. J. 367 (Pt 2), 423–431. 10.1042/BJ20020584 12102654 PMC1222893

[B18] Jaggar J. H. Porter V. A. Lederer W. J. Nelson M. T. (2000). Calcium sparks in Smooth Muscle. Am. J. Physiology-Cell Physiol. 278, C235–C256. 10.1152/ajpcell.2000.278.2.c235 10666018

[B19] Jayasinghe I. Clowsley A. H. Lin R. Lutz T. Harrison C. Green E. (2018). True Molecular Scale Visualization of Variable Clustering Properties of Ryanodine Receptors. Cel Rep. 22 (2), 557–567. 10.1016/j.celrep.2017.12.045 PMC5775502 29320748

[B20] Jones P. P. MacQuaide N. Louch W. E. (2018). Dyadic Plasticity in Cardiomyocytes. Front. Physiol. 9, 1773. 10.3389/fphys.2018.01773 30618792 PMC6298195

[B21] Lanner J. T. Georgiou D. K. Joshi A. D. Hamilton S. L. (2010). Ryanodine Receptors: Structure, Expression, Molecular Details, and Function in Calcium Release. Cold Spring Harbor Perspect. Biol. 2, a003996. 10.1101/cshperspect.a003996 PMC2964179 20961976

[B22] Lewis R. S. (2011). Store-operated Calcium Channels: New Perspectives on Mechanism and Function. Cold Spring Harbor Perspect. Biol. 3 (12), a003970. 10.1101/cshperspect.a003970 PMC3225942 21791698

[B23] Li Z. Liu L. Deng Y. Ji W. Du W. Xu P. (2011). Graded Activation of CRAC Channel by Binding of Different Numbers of STIM1 to Orai1 Subunits. Cell Res 21 (2), 305–315. 10.1038/cr.2010.131 20838418 PMC3193435

[B24] Luik R. M. Wang B. Prakriya M. Wu M. M. Lewis R. S. (2008). Oligomerization of STIM1 Couples ER Calcium Depletion to CRAC Channel Activation. Nature 454 (7203), 538–542. 10.1038/nature07065 18596693 PMC2712442

[B26] Mazel T. Raymond R. Raymond-Stintz M. Jett S. Wilson B. S. (2009). Stochastic Modeling of Calcium in 3D Geometry. Biophysical J. 96, 1691–1706. 10.1016/j.bpj.2008.10.066 PMC2996128 19254531

[B27] McIvor E. Coombes S. Thul R. (2018). Three-dimensional Spatio-Temporal Modelling of Store Operated Ca2+ Entry: Insights into ER Refilling and the Spatial Signature of Ca2+ Signals. Cell calcium 73, 11–24. 10.1016/j.ceca.2018.03.006 29880194

[B28] Mezu-Ndubuisi O. J. Maheshwari A. (2021). The Role of Integrins in Inflammation and Angiogenesis. Pediatr. Res. 89 (7), 1619–1626. 10.1038/s41390-020-01177-9 33027803 PMC8249239

[B29] Okeke E. Dingsdale H. Parker T. Voronina S. Tepikin A. V. (2016). Endoplasmic Reticulum-Plasma Membrane Junctions: Structure, Function and Dynamics. J. Physiol. 594, 2837–2847. 10.1113/jp271142 26939537 PMC4887688

[B30] Okubo Y. Suzuki J. Kanemaru K. Nakamura N. Shibata T. Iino M. (2015). Visualization of Ca2+ Filling Mechanisms upon Synaptic Inputs in the Endoplasmic Reticulum of Cerebellar Purkinje Cells. J. Neurosci. 35, 15837–15846. 10.1523/JNEUROSCI.3487-15.2015 26631466 PMC6605451

[B31] Oloveczky B. Verkman A. (1998). Monte Carlo Analysis of Obstructed Diffusion in Three Dimensions: Applications to Molecular Diffusion in Organelles. Biophysical J. 74, 2722–2730. 10.1016/S0006-3495(98)77978-0 PMC1299612 9591696

[B34] Putney J. W. (2009). Capacitative Calcium Entry: from Concept to Molecules. Immunological Rev. 231 (1), 10–22. 10.1111/j.1600-065x.2009.00810.x 19754887

[B36] Roggenkamp H. G. Khansahib I. Hernandez C L. C. Zhang Y. Lodygin D. Krüger A. (2021). HN1L/JPT2: A Signaling Protein that Connects NAADP Generation to Ca^2+^ Microdomain Formation. Sci. signaling 14 (675), eabd5647. 10.1126/scisignal.abd5647 33758062

[B37] Rückl M. Rüdiger S. (2016). Calcium Waves in a Grid of Clustered Channels with Synchronous IP_3_ Binding and Unbinding. The Eur. Phys. J. E, Soft matter 39 (11), 108. 10.1140/epje/i2016-16108-4 27848113

[B39] Sato D. Bers D. M. (2011). How Does Stochastic Ryanodine Receptor-Mediated Ca Leak Fail to Initiate a Ca Spark. Biophysical J. 101 (10), 2370–2379. 10.1016/j.bpj.2011.10.017 PMC3218344 22098735

[B40] Schaff J. Fink C. C. Slepchenko B. Carson J. H. Loew L. M. (1997). A General Computational Framework for Modeling Cellular Structure and Function. Biophysical J. 73, 1135–1146. 10.1016/s0006-3495(97)78146-3 PMC1181013 9284281

[B42] Solovey G. Fraiman D. Pando B. Ponce Dawson S. (2008). Simplified Model of Cytosolic Ca2+ Dynamics in the Presence of One or Several Clusters of Ca2+ -release Channels. Phys. Rev. E Stat. Nonlin Soft Matter Phys. 78 (4 Pt 1), 041915. 10.1103/PhysRevE.78.041915 18999463

[B43] Stathopulos P. B. Li G.-Y. Plevin M. J. Ames J. B. Ikura M. (2006). Stored Ca2+ Depletion-Induced Oligomerization of Stromal Interaction Molecule 1 (STIM1) via the EF-SAM Region. J. Biol. Chem. 281 (47), 35855–35862. 10.1074/jbc.m608247200 17020874

[B44] Streb H. Irvine R. F. Berridge M. J. Schulz I. (1983). Release of Ca2+ from a Nonmitochondrial Intracellular Store in Pancreatic Acinar Cells by Inositol-1,4,5-Trisphosphate. Nature 306 (5938), 67–69. 10.1038/306067a0 6605482

[B46] Swaminathan D. Ullah G. Jung P. (2009). A Simple Sequential-Binding Model for Calcium Puffs. Chaos 19 (3), 037109. 10.1063/1.3152227 19792034 PMC2826368

[B48] Taylor C. W. Machaca K. (2019). IP3 Receptors and Store-Operated Ca2+ Entry: a License to Fill. Curr. Opin. Cel Biol. 57, 1–7. 10.1016/j.ceb.2018.10.001 30368032

[B49] Thillaiappan N. B. Chavda A. P. Tovey S. C. Prole D. L. Taylor C. W. (2017). Ca2+ Signals Initiate at Immobile IP3 Receptors Adjacent to ER-Plasma Membrane Junctions. Nat. Commun. 8 (1), 1505. 10.1038/s41467-017-01644-8 29138405 PMC5686115

[B51] Thul R. Thurley K. Falcke M. (2009). Toward a Predictive Model of Ca2+ Puffs. Chaos 19 (3), 037108. 10.1063/1.3183809 19792033

[B52] Trebak M. Kinet J.-P. (2019). Calcium Signalling in T Cells. Nat. Rev. Immunolimmunology 19 (3), 154–169. 10.1038/s41577-018-0110-7 PMC6788797 30622345

[B53] Walker M. A. Gurev V. Rice J. J. Greenstein J. L. Winslow R. L. (2017). Estimating the Probabilities of Rare Arrhythmic Events in Multiscale Computational Models of Cardiac Cells and Tissue. Plos Comput. Biol. 13 (11), e1005783. 10.1371/journal.pcbi.1005783 29145393 PMC5689829

[B55] Wolf I. M. Diercks B. P. Gattkowski E. Czarniak F. Kempski J. Werner R. (2015). Frontrunners of T Cell Activation: Initial, Localized Ca2+ Signals Mediated by NAADP and the Type 1 Ryanodine Receptor. Sci. Signal. 8 (398), ra102. 10.1126/scisignal.aab0863 26462735

[B57] Wu M. M. Buchanan J. Luik R. M. Lewis R. S. (2006). Ca2+ Store Depletion Causes STIM1 to Accumulate in ER Regions Closely Associated with the Plasma Membrane. J. Cel. Biol. 174 (6), 803–813. 10.1083/jcb.200604014 PMC2064335 16966422

[B58] Yin C.-C. D’Cruz L. G. Lai F. A. (2008). Ryanodine Receptor Arrays: Not Just a Pretty Pattern. Trends Cell Biology 18 (4), 149–156. 10.1016/j.tcb.2008.02.003 18329877

