# Supplementary material for: Three-Dimensional Model of Sub-Plasmalemmal Ca2+ Microdomains Evoked by T Cell Receptor/CD3 Complex Stimulation
Source: Front Mol Biosci. 2022 Feb 23;9:811145. doi: 10.3389/fmolb.2022.811145 (PMC8906516; doi:10.3389/fmolb.2022.811145)
Supplement: Supplementary file 1 [file Presentation1.pdf]

Supplementary Information

1. Description of the mathematical model.

The mathematical model used in this study is the same as in Gil et al. (2021), except that we consider Ca^{2+} release through RYR instead of IP_3R, and consider the presence of STIM1 and STIM2 instead of only STIM2. The model describes the evolution in time and space of free cytosolic Ca^{2+} concentration $C_C= C_C(t,x,y,z)$ and of free ER Ca^{2+} concentration $C_S= C_S(t,x,y,z)$, both in close proximity of the ER-PM junction. Changes in C_C and C_S are governed by the diffusion equations S1 and S2 respectively,

$$\frac{\partial C_C}{\partial t} - D_C \nabla^2 C_C = 0 \qquad \text{(S1)},$$

$$\frac{\partial C_S}{\partial t} - D_S \nabla^2 C_S = 0 \qquad \text{(S2)},$$

together with the initial conditions $C_{C,0}$ and $C_{S,0}$:

$$C_{C,0} = 0.03\ \mu M \qquad \text{(S3)},$$

$$C_{S,0} = 400\ \mu M \qquad \text{(S4)}.$$

Ca^{2+} ions are transferred across domains through channels and pumps. The corresponding boundary conditions follow:

For C_C,

$$D_C \left.\frac{\partial C_C}{\partial z}\right|_{z=500nm} = \begin{cases} J_{ORAI}, & \text{at ORAI1 channels} \\ 0, & \text{rest of the PM,} \end{cases} \qquad \text{(S5)},$$

$$D_C \left.\frac{\partial C_C}{\partial z}\right|_{z=485nm} = \begin{cases} J_{SERCA}, & \text{at SERCA pumps} \\ 0, & \text{rest of the ERM,} \end{cases} \qquad \text{(S6)},$$

$$D_C \left.\frac{\partial C_C}{\partial \phi}\right|_{\phi=\frac{5\pi}{6}} = \begin{cases} J_{RYR}, & \text{at RYR channels} \\ 0, & \text{rest of the sub} - \text{PM ER surface ,} \end{cases} \qquad \text{(S7)},$$

$$C_C|_{x,y\, \in \{0,400\}nm} = C_{C,0}. \qquad \text{(S8)}.$$

For C_S,

$$D_S \frac{\partial C_S}{\partial z}\bigg|_{z=485nm} = \begin{cases} J_{SERCA}, & \text{at SERCA pumps} \\ 0, & \text{rest of the ERM,} \end{cases} \quad \text{(S9),}$$

$$D_S \frac{\partial C_S}{\partial \phi}\bigg|_{\phi=\frac{5\pi}{6}} = \begin{cases} J_{RYR}, & \text{at RYR channels} \\ 0, & \text{rest of the sub} - \text{PM ER surface ,} \end{cases} \quad \text{(S10),}$$

$$C_S|_{z=0} = C_{S,0}. \quad \text{(S11).}$$

The influx across one ORAI1 channel per unit area (Eq. S12) follows Faraday's law with the magnitude of a single channel current *I_{ORAI} = 2.1 fA,* determined experimentally by Hoth & Penner (1992), *F* the Faraday constant, *z the* charge of a Ca^{2+} ion and the surface of the channel pore *A_O = 0.25 nm^2* measured by Parekh & Putney (2005)

$$J_{ORAI} = \frac{I_{ORAI}}{F \cdot z \cdot A_o} \cdot f_i\left(C_S^{loc}\right) \cdot \phi_{ORAI} \quad \text{(S12),}$$

multiplied by $f_i\left(C_S^{loc}\right)$, a function of the average local concentration of luminal Ca^{2+} around the pore of the RYR (Eq. S13), computed in a 108 nm^3 volume surrounding each RYRs at the side of the ER. In the simulations, this concentration determines the level of ORAI1 channel activation, since it was suggested by Li et al. (2011) that this activation is not an "all or none" phenomenon but rather a graded process of four conductance states, which depends on the amount of bounded STIM proteins determined by the level of ER depletion. We consider two situations: one in which a STIM2 homotetramer is bound to ORAI1 and one in which a STIM2 dimer and a STIM1 dimer are bound to ORAI. These cases are symbolised by *i*=1 and *i*=1/2. Functions f_i are given by

$$f_2\left(C_S^{loc}\right) = \begin{cases} 0.07, & C_S^{loc} > 450\,\mu M \\ 0.21, & 450\,\mu M \leq C_S^{loc} < 334\,\mu M \\ 0.54, & 334\,\mu M \leq C_S^{loc} < 250\,\mu M \\ 1, & C_S^{loc} \geq 250\,\mu M \end{cases} \quad \text{(S13),}$$

$$f_{1/2}\left(C_S^{loc}\right) = \begin{cases} 0.07, & C_S^{loc} > 340\,\mu M \\ 0.21, & 340\,\mu M \leq C_S^{loc} < 260\,\mu M \\ 0.54, & 260\,\mu M \leq C_S^{loc} < 197\,\mu M \\ 1, & C_S^{loc} \geq 197\,\mu M \end{cases} \quad \text{(S14).}$$

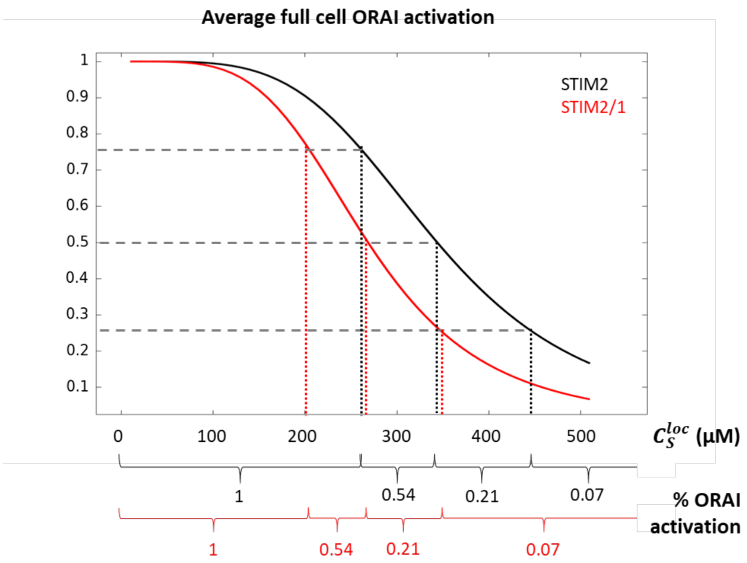

Figure S1.

Given that we do not consider Ca^{2+}-bound STIM diffusion explicitly, we define the ranges of local luminal Ca^{2+} that correspond to each ORAI1 activation level. This assumption follows the observation of pre-formed clusters of ORAI1/STIM2 and ORAI1/STIM1/STIM2 (Diercks et al., 2018 and Weiss, unpublished), allowing to consider that SOCE is immediately activated after Ca^{2+} unbinding from STIM2 homotetramers or STIM1/2 heterotetramers. The function $f_2\left(C_S^{loc}\right)$ defined in (S13) is the same as in Gil et al. (2021). It follows a Hill function corresponding to SOC activation by STIM2 homotetramers (Fig. S1, black) with K_{D2} = 334 μM, which is twice the STIM1's dissociation constant observed experimentally by Luik et al. (2008), taking into account STIM2's lower Ca^{2+} affinity (Luik et al., 2008; Brandman et al., 2007). In the case of SOC activation by STIM1/2 heterotetramers (S14), the function $f_{1/2}\left(C_S^{loc}\right)$ follows the fitted Hill function (Fig. S1, red),

$$\frac{(K_{D2})^2 * (K_{D1})^2}{(K_{D2})^2 * (K_{D1})^2 + {C_S^{loc}}^4} \quad \text{(S15).}$$

According to Subedi et al. (2018), STIM1 dissociation constant increases under the effect of STIM2, thus we propose K_{D1} = 200.4 μM instead of 167 μM that corresponds to STIM1 homotetramers (Luik et al. 2008).

An additional function Φ_{ORAI} multiplies the influx through one ORAI channel. It allows to simulate the two configurations of the model. The first one in which the RYR are closed and Ca^{2+} enters the cytosol only across ORAI1 channels and the second one where we consider open RYR,

$$\phi_{ORAI} = \begin{cases} \Pi(t)_i, & \text{closed RYR} \\ 1, & \text{open RYR} \end{cases} \quad \text{(S16).}$$

In a similar way, the influx across one RYR is given by Eq. S17, with a single current magnitude of $I_{RYR} = 0.35\ pA$ as estimated by Guo et al. (2012) at resting Ca^{2+} concentrations in the cytosol and in the ER and $A_{RYR} = 0.41\ nm^2$ as stated in Thul & Falcke (2004),

$$J_{RYR} = \frac{I_{RYR}}{F \cdot z \cdot A_{RYR}} \cdot \frac{(C_s - C_C)}{(C_{s,0} - C_{C,0})} \cdot \phi_{RYR} \qquad \text{(S17).}$$

The concentrations difference $(C_S - C_C)$ is computed locally at each RYR and $(C_{S,0} - C_{C,0})$ stand for the difference in resting concentrations. Given the time scales of the simulations, buffering is not considered. Once more we multiply the flux by a Φ_{RYR} function (S18), which follows our two configurations,

$$\phi_{RYR} = \begin{cases} 0, & \text{closed RYR} \\ \Pi(t)_m, & \text{open RYR} \end{cases} \qquad \text{(S18).}$$

The rectangular function $\Pi(t)$ changes in time between 0 and 1 deterministically and controls whether a channel is open or closed, accordingly with the respective configuration,

$$\Pi(t)_{\{i,m\}} = \Pi\left(\frac{t - \tau_{c_{\{i,m\}}}}{\tau_d}\right) \qquad \text{(S19),}$$

with $i \in \{1,2,3,4,5\}$ the corresponding ORAI1 channel and $m \in \{1 \ldots 16\}$ the corresponding RYR depending on the configuration. The channels open during a time $\tau_d = 0.064\ s$. The central time $\tau_{c_{\{i,m\}}}$ when the channels are open is given by the following sequence:

$$\tau_{c\{i,m\}_n} = n * 0.1 + (n - 0.5) * 0.064 \quad \forall\, n \geq \{i,m\},\ \ n > 0 \qquad \text{(S20),}$$

where n increases gradually and represents the number of open channels (ORAI1 or RYR). τ_c is expressed in seconds.

We use bi-directional SERCA2b pumps as done by Shannon et al. (2004) with the respective kinetics parameters taken from Lytton et al. (1992), as done in McIvor et al. (2018),

$$J_{SERCA} = \frac{Q}{A_S} * V_{max} * \left[\frac{\left(\frac{C_C}{K_F}\right)^{H2b} - \left(\frac{C_S}{K_R}\right)^{H2b}}{1 + \left(\frac{C_C}{K_F}\right)^{H2b} + \left(\frac{C_S}{K_R}\right)^{H2b}} \right] \quad \text{(S21)},$$

a summary of all parameters is given in Table S1.

We assume the evolution of Ca^{2+} concentration to be governed purely by isotropic diffusion, therefore we use an unbuffered cytosolic diffusion D_J=220 um^2/s (McIvor et al., 2018; Samanta et al., 2015; Hogan, 2015) considering that the high Ca^{2+} concentration at the mouth of the channels will saturate local Ca^{2+} buffers (Parekh, 2008). The exact value of the Ca^{2+} diffusion coefficient in the ER lumen remains yet to be determined, although it is known to be smaller than in the cytosol as a result of its irregular and cramped shape and the high concentration of buffers. A luminal diffusion coefficient including the effect of buffers has been measured to be around D_S=10 um^2/s (Dayel et al., 1999; Swietach et al., 2008; McIvor et al., 2018), a smaller diffusion being possibly associated to the more tubular part.

2. Analysis of the junctional Ca^{2+} amplitude (C_C) when the surrounding RYR cluster sits 45 nm away from the junction, half the original distance.

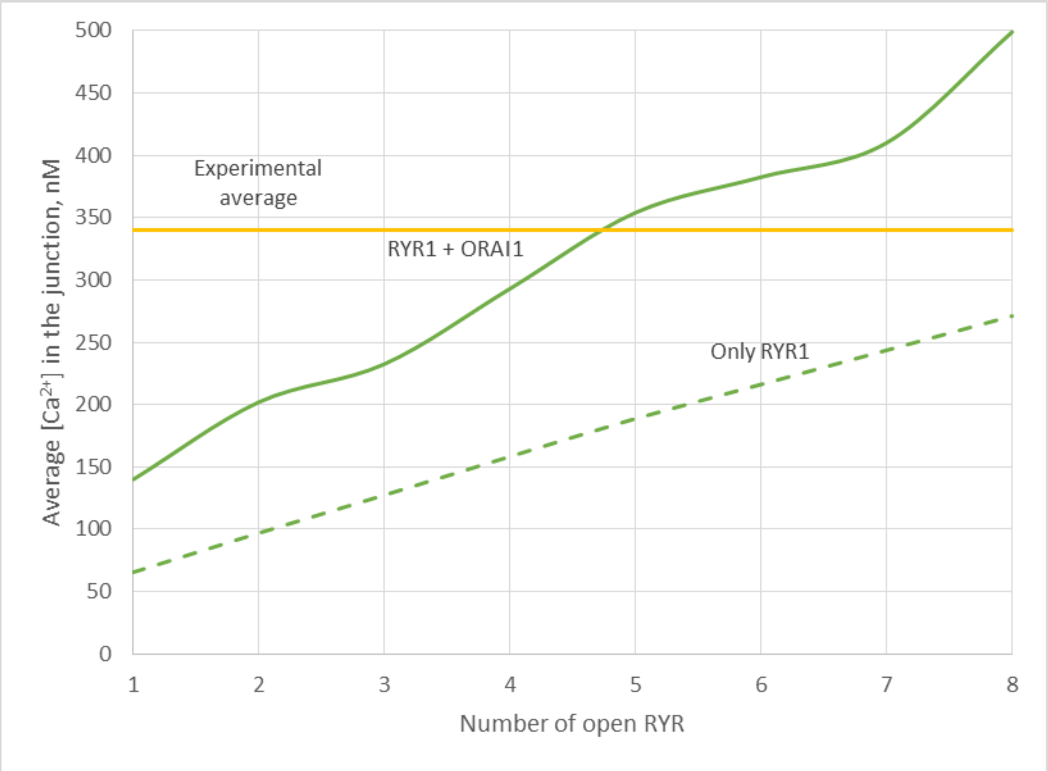

Figure S2.

Evolution of the amplitude of the simulated Ca^{2+} microdomains with the number of simultaneously open RYRs in the junction, showing that experimentally observed microdomains can in principle result from the opening of ORAI1 channels induced by the spontaneous opening of a few RYRs near the junction as a result of local depletion of ER Ca^{2+}, in conditions of a full ER. Dotted line represents junctional Ca^{2+} concentration reached in the absence of ORAI1 channels. In principle, when the distance of the RYR cluster to the junction is reduced in half from 90 nm to 45 nm, opening of 5 RYRs simultaneously (2 less than with 90 nm) is already sufficient to simulate Ca^{2+} microdomains observed experimentally.

3. **Analysis of the frequency of simultaneously open IP_3R in a cluster of 8 receptors during a puff.**

In Figure 8, we analyse the number of simultaneously open RYRs in the cluster considering their mean open and closed time during activity. Here, we perform the same analysis for a cluster of IP_3R's in conditions corresponding to non-TCR/CD3 dependent microdomains arising in T cells. These microdomains rely on IP3 signaling and subsequent SOCE via preformed ORAI1/STIM2 complexes. During a puff, the single receptor open probability is 0.2 (Bezprozvanny et al., 1991) with a mean open time of 5ms (Rahman and Taylor, 2009) at high IP_3 and Ca^{2+} concentrations. As shown in Figure S2, in these conditions, most of the time 2 IP_3R's are open simultaneously. This is in qualitative agreement with the spatio-temporal simulations of Gil et al. (2021), which indicate that non TCR/CD3 dependent Ca^{2+} microdomains rely on the simultaneous opening of 3-4 IP_3R's located around the junction. The resulting local depletion in ER Ca^{2+} triggers the opening of ORAI1 channels bound to STIM2 homotetramers.

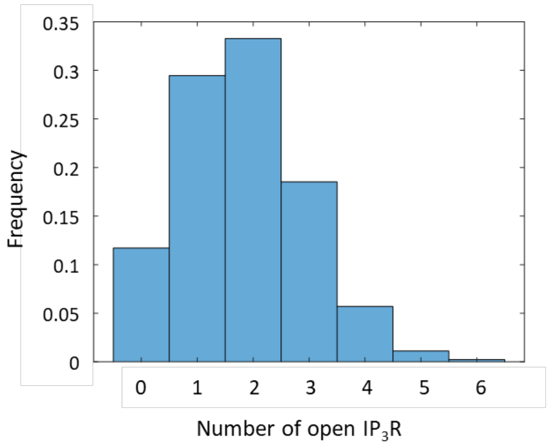

Figure S3.

4. **List of parameter values used in the spatio-temporal simulations of Ca^{2+} microdomains**

Table S1

Parameter	*Value*	*Unit*	*Description*	*Reference*
H_J	15	nm	Height ER-PM junction	Wu et al., 2006
H_S	485	nm	Height sub-PM ER	McIvor et al., 2018
r1	200	nm	Bottom radius sub-PM ER	
r2	100	nm	Top radius sub-PM ER	Samanta et al. 2015
r3	40	nm	Radius ORAI ring	Samanta et al. 2015
r4	30	nm	Distance ORAI to SERCA ring	
$C_{C,0}$	30	nM	Cytosol basal concentration	Diercks et al., 2018
$C_{S,0}$	400	µM	sub-PM ER initial concentration	Lewis, 2011
D_C	220	um^2/s	Diffusion coefficient cytosol	Hogan, 2015
D_S	10	um^2/s	Diffusion coefficient ER	Swietach et al., 2008
F	96485	C/mol	Faraday's constant	
z	2		Valency of Ca^{2+} ions	
A_O	0.25	nm^2	Area ORAI channel	Parekh & Putney 2005
I_{ORAI}	2.1	fA	ORAI single channel current	Zweifach & Lewis, 1993
A_{RYR}	0.41	nm^2	Area RYR	Thul & Falcke, 2004
I_{RYR}	0.35	pA	RYR single channel current	Guo et al., 2012
A_S	0.98	nm^2	Area SERCA pump	
K_F	0.27	µM	SERCA2b pump Ca^{2+} affinity	Lytton et al. 1992
K_R	1700	µM	SERCA pump Ca^{2+} affinity in reverse mode	Shannon et al. 2004
H2b	1.7		Hill coefficient SERCA2b	Lytton et al. 1992
$V_{\mathbf{max}}$	6.0E-23	mol/s	Maximal SERCA2b pump rate	Lytton et al. 1992
Q	2.6		Temperature coefficient	Shannon et al. 2004
τ_d	0.065	s	Duration of early NAADP-dependent microdomains	Estimated based on Diercks et al., 2018
T	1.5	s	Final simulation time	
K_{D2}	334 = 2*167	µM	Ca^{2+} dissociation constant of STIM2	Estimated based on Luik et al. 2008 (see text)

K_{D1}	200.4 = 1.2*167	µM	Ca^{2+} dissociation constant of STIM1	Estimated based on Luik et al. 2008 and Subedi et al. 2018 (see text)

Animations

All animations can be found at:

https://www2.ulb.ac.be/sciences/utc/animations/animations3.html

Animation S1a. Simulated time evolution of the *cross-sectional* Ca^{2+} profiles along the z-axis resulting from the opening of 1 to 9 RYRs inside the junction, which in turn induces the opening of ORAI1 channels in the junctions as a result of local depletion of ER Ca^{2+}. Results obtained with the default value for Ca^{2+} diffusion coefficient in the ER ($D_S = 10\ \mu m^2/s$). Left bar indicates the colour code, together with the minimal and maximal concentration reached. RYRs open during 64 ms. Experimentally observed microdomains do not agree with the opening of the RYRs inside the junction given the low contribution of the opening of the ORAI1 in conditions of a full ER.

Animation S1b. Simulated time evolution of Ca^{2+} profiles *in the junction* resulting from the opening of 1 to 9 of the RYRs inside the junction, which in turn induces the opening of ORAI1 channels in the junctions as a result of local depletion of ER Ca^{2+}. Results obtained with the default value for Ca^{2+} diffusion coefficient in the ER ($D_S = 10\ \mu m^2/s$). Left bar indicates the colour code, together with the minimal and maximal concentration reached. RYRs open during 64 ms. Upon depletion of local Ca^{2+} in the ER, which is quasi-instantaneous, ORAI1 channels open in different states to an extent that depends on this local concentration, as defined by the function *f* (see Supplementary information). ORAI1 opening is assumed to occur immediately after depletion because ORAI1-STIM2-STIM1 aggregates are pre-formed (Diercks et al, 2018).

Animation S2a. Simulated time evolution of the *cross-sectional* Ca^{2+} profiles along the z-axis resulting from the opening of 1 to 8 RYRs adjacent to the junction simultaneously, which in turn induces the opening of ORAI1 channels in the junctions as a result of local depletion of ER Ca^{2+}. Results obtained with the default value for Ca^{2+} diffusion coefficient in the ER ($D_S = 10\ \mu m^2/s$). Left bar indicates the colour code, together with the minimal and maximal concentration reached. RYRs open during 64 ms. Experimentally observed microdomains can

in principle result from the opening of ORAI1 channels induced by the spontaneous opening of a few RYRs near the junction, in conditions of a full ER.

Animation S2b. Simulated time evolution of the *cross-sectional* Ca^{2+} profiles along the z-axis resulting from the opening of 1 to 8 RYRs adjacent to the junction simultaneously, in the absence of ORAI1 channels. Results obtained with the default value for Ca^{2+} diffusion coefficient in the ER ($D_S = 10\ \mu m^2/s$). Left bar indicates the colour code, together with the minimal and maximal concentration reached. RYRs open during 64 ms. Experimentally observed microdomains cannot result from the spontaneous opening of a few RYRs near the junction in the absence of ORAI1 channels, in conditions of a full ER.

Animation S2c. Simulated time evolution of the three-dimensional Ca^{2+} profiles in the junction and Sub-PM ER resulting from the opening of 1 to 8 of the RYRs. Ca^{2+} profiles in the junction, in the cytosol adjacent to the junction and in the sub-PM ER with the default value for Ca^{2+} diffusion coefficient in the ER ($D_S = 10\ \mu m^2/s$). Local depletion of ER Ca^{2+} provokes the opening of the nearby ORAI1s. This situation corresponds to the one shown in Animation S2a,d. The right bar indicates the colour code of Ca^{2+} concentration in the cytosol while the left bar indicates the colour code of Ca^{2+} concentration in the ER.

Animation S2d. Simulated time evolution of the three-dimensional Ca^{2+} profiles in the whole geometry resulting from the opening of 1 to 8 of the RYRs. Ca^{2+} profiles in the junction, in the cytosol adjacent to the junction and in the sub-PM ER with the default value for Ca^{2+} diffusion coefficient in the ER ($D_S = 10\ \mu m^2/s$). Local depletion of ER Ca^{2+} provokes the opening of the nearby ORAI1s. This situation corresponds to the one shown in Animation S2a,c. The right bar indicates the colour code of Ca^{2+} concentration in the cytosol while the left bar indicates the colour code of Ca^{2+} concentration in the ER.

Animation S3. Simulated time evolution of Ca^{2+} profiles *in the junction* resulting from the opening of 1 to 9 of the RYRs adjacent to the junction simultaneously, which in turn induces the opening of ORAI1 channels in the junctions as a result of local depletion of ER Ca^{2+}. Results obtained with the default value for Ca^{2+} diffusion coefficient in the ER ($D_S = 10\ \mu m^2/s$). Left bar indicates the colour code, together with the minimal and maximal concentration reached. RYRs open during 64 ms. This situation corresponds to the one shown in Animation S2a,c,d. Upon depletion of local Ca^{2+} in the ER, which is quasi-instantaneous, ORAI1 channels open in different states to an extent that depends on this local concentration, as defined by the

function *f* (see Supplementary information). ORAI1 opening is assumed to occur immediately after depletion because ORAI1-STIM2-STIM1 aggregates are pre-formed (Diercks et al, 2018).

Animation S4. Simulated time evolution of the *cross-sectional* Ca^{2+} profiles along the z-axis resulting from the opening of 1 to 16 RYRs adjacent to the junction simultaneously, which in turn induces the opening of ORAI1 channels in the junctions as a result of local depletion of ER Ca^{2+}. Results obtained with the default value for Ca^{2+} diffusion coefficient in the ER ($D_S = 10\ \mu m^2/s$). Left bar indicates the colour code, together with the minimal and maximal concentration reached. RYRs open during 64 ms. The second cluster of 8 RYRs is located directly underneath the first cluster. At 8 simultaneously open RYRs, the complete cluster of 5 ORAI1 channels reach their maximum open state possible, in conditions of a full ER.

Animation S5a. Simulated time evolution *in the junction* of the most probable Ca^{2+} microdomains resulting from a T cell transition between quiescent to early activation, accordingly with Fig 8. Results obtained with the default value for Ca^{2+} diffusion coefficient in the ER ($D_S = 10\ \mu m^2/s$). Left bar indicates the colour code, together with the minimal and maximal concentration reached. Initial, non TCR/CD3-dependent Ca^{2+} microdomains formed by the opening of 2 IP_3Rs adjacent to the junction and further opening of ORAI1 channels bound to STIM2/2. Followed by basal opening of five ORAI1 channels, one inherently co-localized with STIM2/2 and four inherently co-localized with STIM2/1 leading to small microdomains arising from nano-scale [Ca^{2+}] fluctuations in the sub-PM ER. Artificial construction. Leading to TCR/CD3-dependent Ca^{2+} microdomains formed by the opening of 6 RYRs adjacent to the junction and further opening of ORAI1 channels bound to STIM2/1. Upon depletion of local Ca^{2+} in the ER, which is quasi-instantaneous, ORAI1 channels open in different states to an extent that depends on this local concentration, as defined by the function *f* (see Supplementary information). ORAI1 opening is assumed to occur immediately after depletion because ORAI1-STIM2-STIM1 aggregates are pre-formed (Diercks et al, 2018).

Animation S5b. Simulated time evolution in the junction and Sub-PM ER of the most probable Ca^{2+} microdomains resulting from a T cell transition between quiescent to early activation, accordingly with Fig 8. Results obtained with the default value for Ca^{2+} diffusion coefficient in the ER ($D_S = 10\ \mu m^2/s$). Left bar indicates the colour code, together with the minimal and maximal concentration reached. This situation corresponds to the one shown in Animation S5a,c. Initial, non TCR/CD3-dependent Ca^{2+} microdomains formed by the opening of 2 IP_3Rs adjacent to the junction and further opening of ORAI1 channels bound to STIM2/2. Followed

by basal opening of five ORAI1 channels, one inherently co-localized with STIM2/2 and four inherently co-localized with STIM2/1 leading to small microdomains arising from nano-scale [Ca^{2+}] fluctuations in the sub-PM ER. Artificial construction. Leading to TCR/CD3-dependent Ca^{2+} microdomains formed by the opening of 6 RYRs adjacent to the junction and further opening of ORAI1 channels bound to STIM2/1.

Animation S5c. Simulated time evolution of the *cross-sectional* Ca^{2+} profiles along the z-axis of the most probable Ca^{2+} microdomains resulting from a T cell transition between quiescent to early activation, accordingly with Fig 8. Results obtained with the default value for Ca^{2+} diffusion coefficient in the ER ($D_S = 10\ \mu m^2/s$). Left bar indicates the colour code, together with the minimal and maximal concentration reached. This situation corresponds to the one shown in Animation S5a,b. Initial, non TCR/CD3-dependent Ca^{2+} microdomains formed by the opening of 2 IP_3Rs adjacent to the junction and further opening of ORAI1 channels bound to STIM2/2. Followed by basal opening of five ORAI1 channels, one inherently co-localized with STIM2/2 and four inherently co-localized with STIM2/1 leading to small microdomains arising from nano-scale [Ca^{2+}] fluctuations in the sub-PM ER. Artificial construction. Leading to TCR/CD3-dependent Ca^{2+} microdomains formed by the opening of 6 RYRs adjacent to the junction and further opening of ORAI1 channels bound to STIM2/1.

References

Bezprozvanny, I., Watras, J., & Ehrlich, B. E. (1991). Bell-shaped calcium-response curves of Ins(1,4,5)P_3- and calcium-gated channels from endoplasmic reticulum of cerebellum. *Nature*, *351*(6329), 751–754.

Dayel, M. J., Hom, E. F., & Verkman, A. S. (1999). Diffusion of green fluorescent protein in the aqueous-phase lumen of endoplasmic reticulum. *Biophysical journal*, *76*(5), 2843–2851.

Hoth, M., & Penner, R. (1992). Depletion of intracellular calcium stores activates a calcium current in mast cells. *Nature*, *355*(6358), 353–356.

Lytton, J., Westlin, M., Burk, S. E., Shull, G. E., & MacLennan, D. H. (1992). Functional comparisons between isoforms of the sarcoplasmic or endoplasmic reticulum family of calcium pumps. *The Journal of biological chemistry*, *267*(20), 14483–14489.

Parekh A. B. (2008). Ca^{2+} microdomains near plasma membrane Ca^{2+} channels: impact on cell function. *The Journal of physiology*, *586*(13), 3043–3054.

Rahman, T., & Taylor, C. W. (2009). Dynamic regulation of IP_3 receptor clustering and activity by IP_3. *Channels (Austin, Tex.)*, *3*(4), 226–232.

Samanta, K., Kar, P., Mirams, G. R., & Parekh, A. B. (2015). Ca^{2+} Channel Re-localization to Plasma-Membrane Microdomains Strengthens Activation of Ca^{2+}-Dependent Nuclear Gene Expression. *Cell reports*, *12*(2), 203–216.

Shannon, T. R., Wang, F., Puglisi, J., Weber, C., & Bers, D. M. (2004). A mathematical treatment of integrated Ca dynamics within the ventricular myocyte. *Biophysical journal*, *87*(5), 3351–3371.

Subedi, K. P., Ong, H. L., Son, G. Y., Liu, X., & Ambudkar, I. S. (2018). STIM2 Induces Activated Conformation of STIM1 to Control Orai1 Function in ER-PM Junctions. *Cell reports*, *23*(2), 522–534.

Swietach, P., Spitzer, K. W., & Vaughan-Jones, R. D. (2008). Ca^{2+}-mobility in the sarcoplasmic reticulum of ventricular myocytes is low. *Biophysical journal*, *95*(3), 1412–1427.

Thul, R., & Falcke, M. (2004). Release currents of IP_3 receptor channel clusters and concentration profiles. *Biophysical journal*, *86*(5), 2660–2673.

Zweifach, A., & Lewis, R. S. (1993). Mitogen-regulated Ca^{2+} current of T lymphocytes is activated by depletion of intracellular Ca^{2+} stores. *Proceedings of the National Academy of Sciences of the United States of America*, *90*(13), 6295–6299.
